# Supplementary material for: Establishment of a Cell Suspension Culture of Ageratina pichinchensis (Kunth) for the Improved Production of Anti-Inflammatory Compounds
Source: Plants (Basel). 2020 Oct 21;9(10):1398. doi: 10.3390/plants9101398 (PMC7594056; doi:10.3390/plants9101398)
Supplement: Supplementary file 1 [file plants-09-01398-s001.zip › plants-921709-supplementary.pdf]

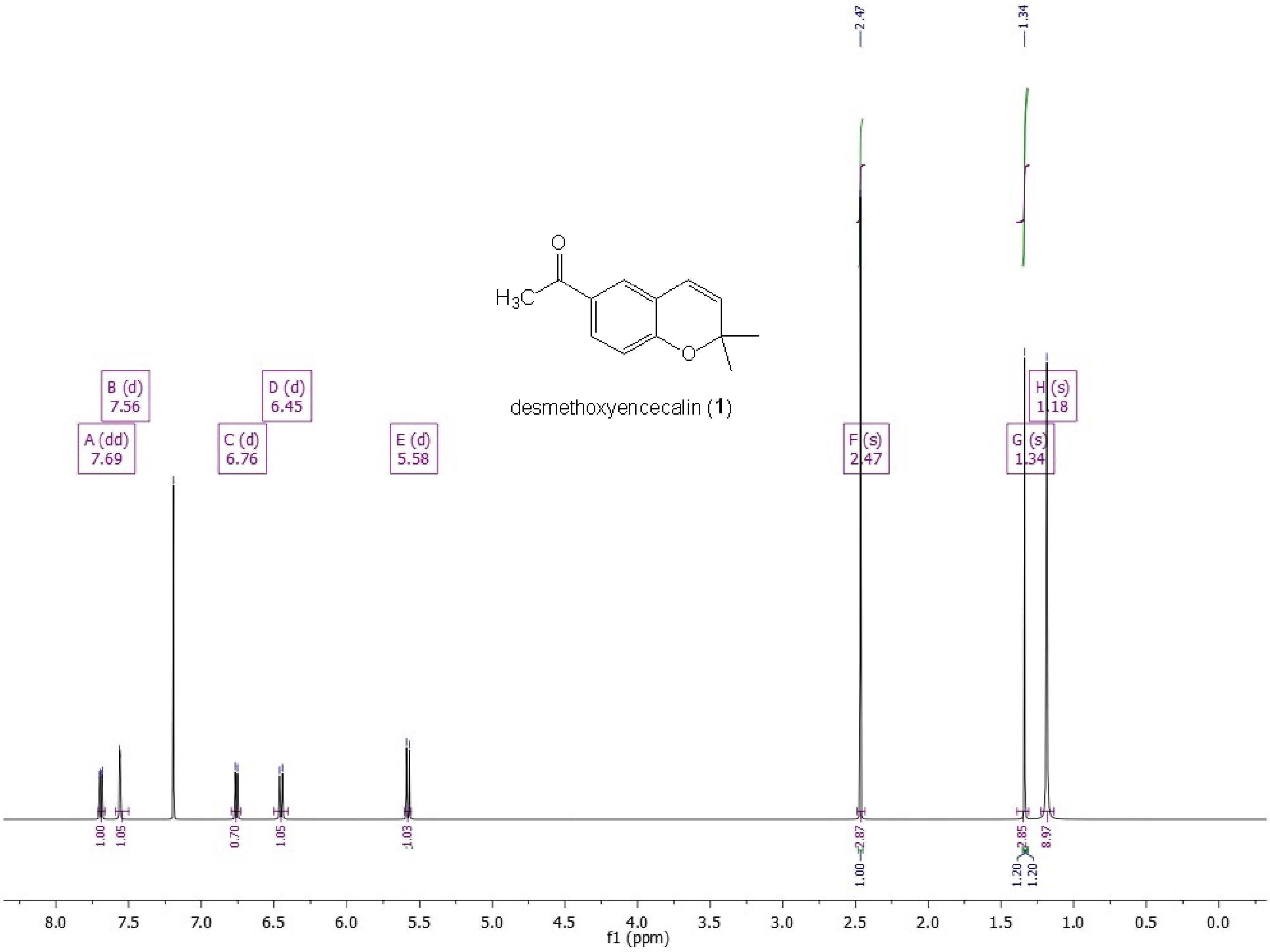

Figure S1. ^{1}H-NMR spectrum (200 MHz, $CDCl_3$) of desmethoxyencecalin (1).

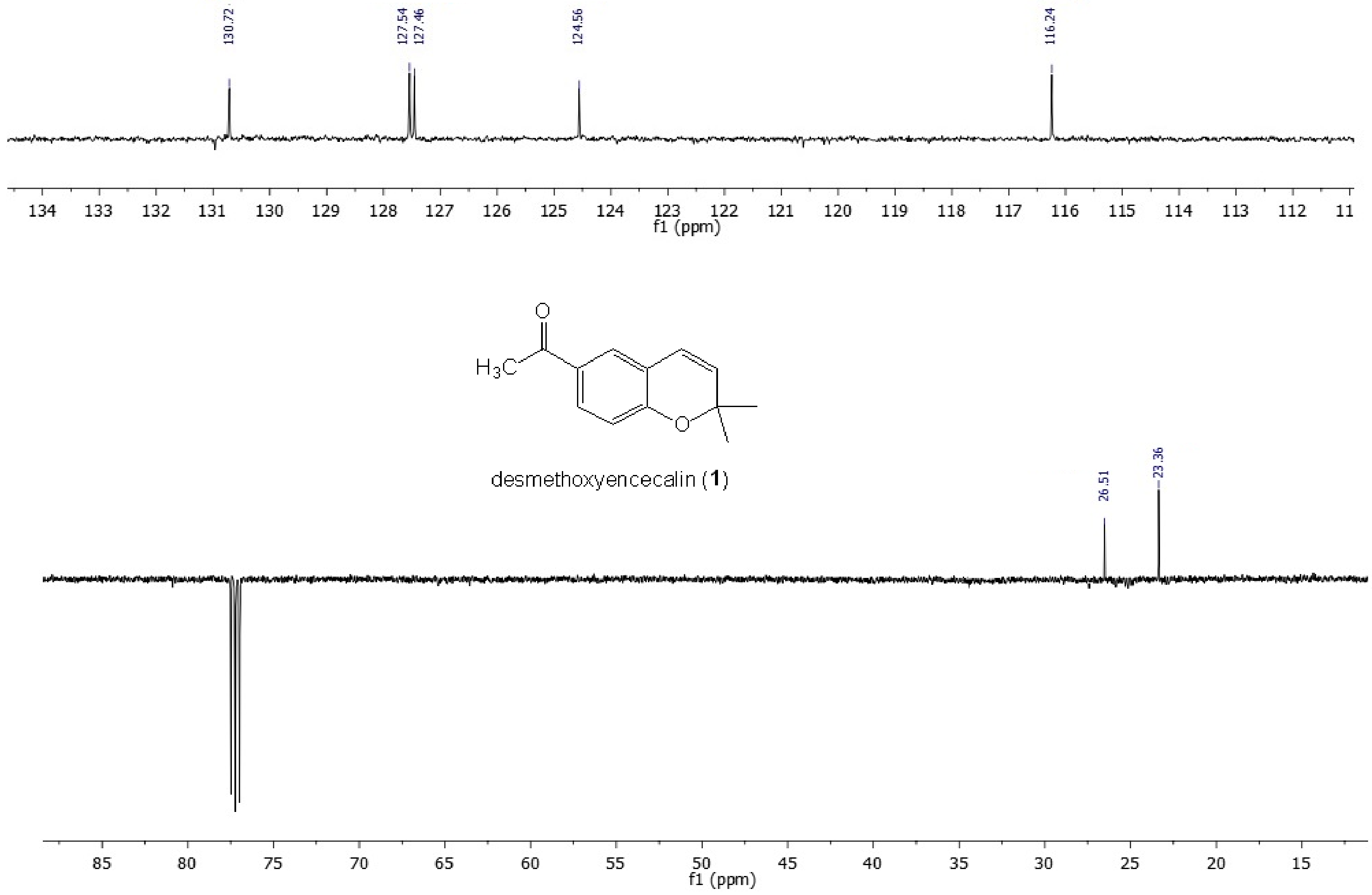

Figure S2. ^{13}C-DEPT-NMR spectrum (50 MHz, $CDCl_3$) of desmethoxyencecalin (1).

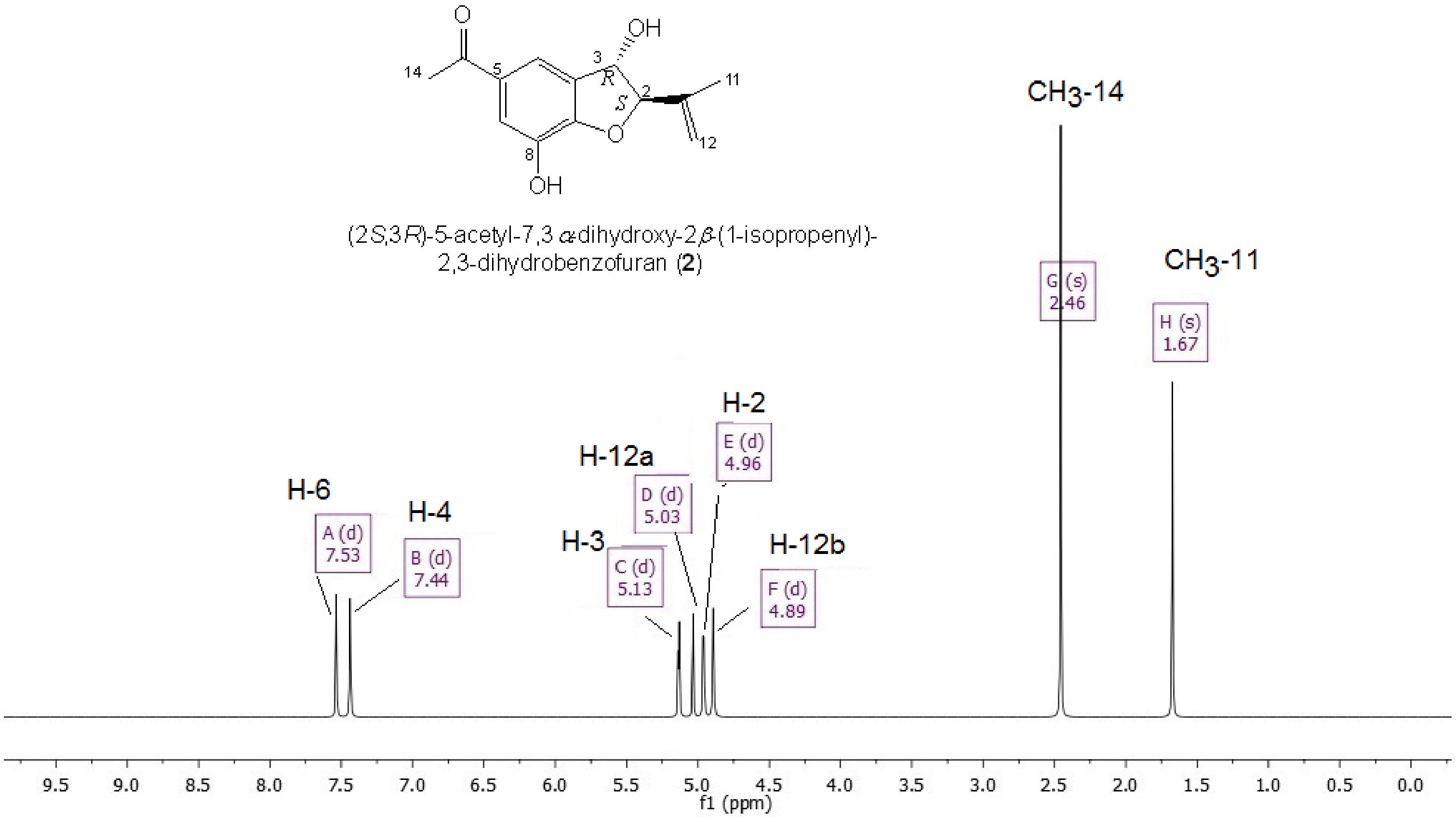

Figure S3. ^{1}H-NMR spectrum (200 MHz, $CDCl_3$: CD_3OD) of (2*S*,3*R*)-5-acetyl-7,3α-dihydroxy-2*β*-(1-isopropenyl)-2,3-dihydrobenzofuran (2).

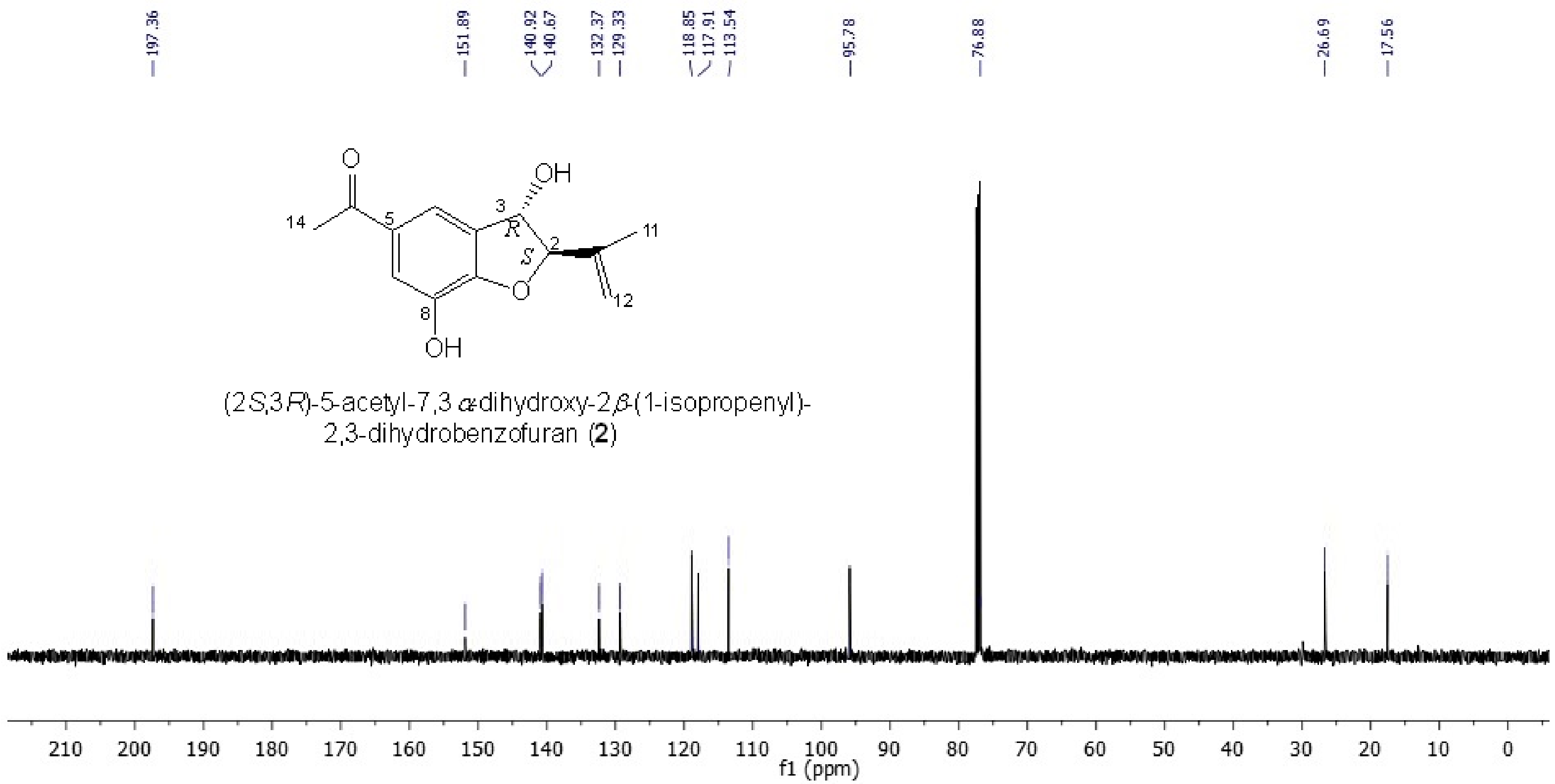

Figure S4. ^{13}C-NMR spectrum (50 MHz, $CDCl_3$: CD_3OD) of (2*S*,3*R*)-5-acetyl-7,3α-dihydroxy-2β-(1-isopropenyl)-2,3-dihydrobenzofuran (2).

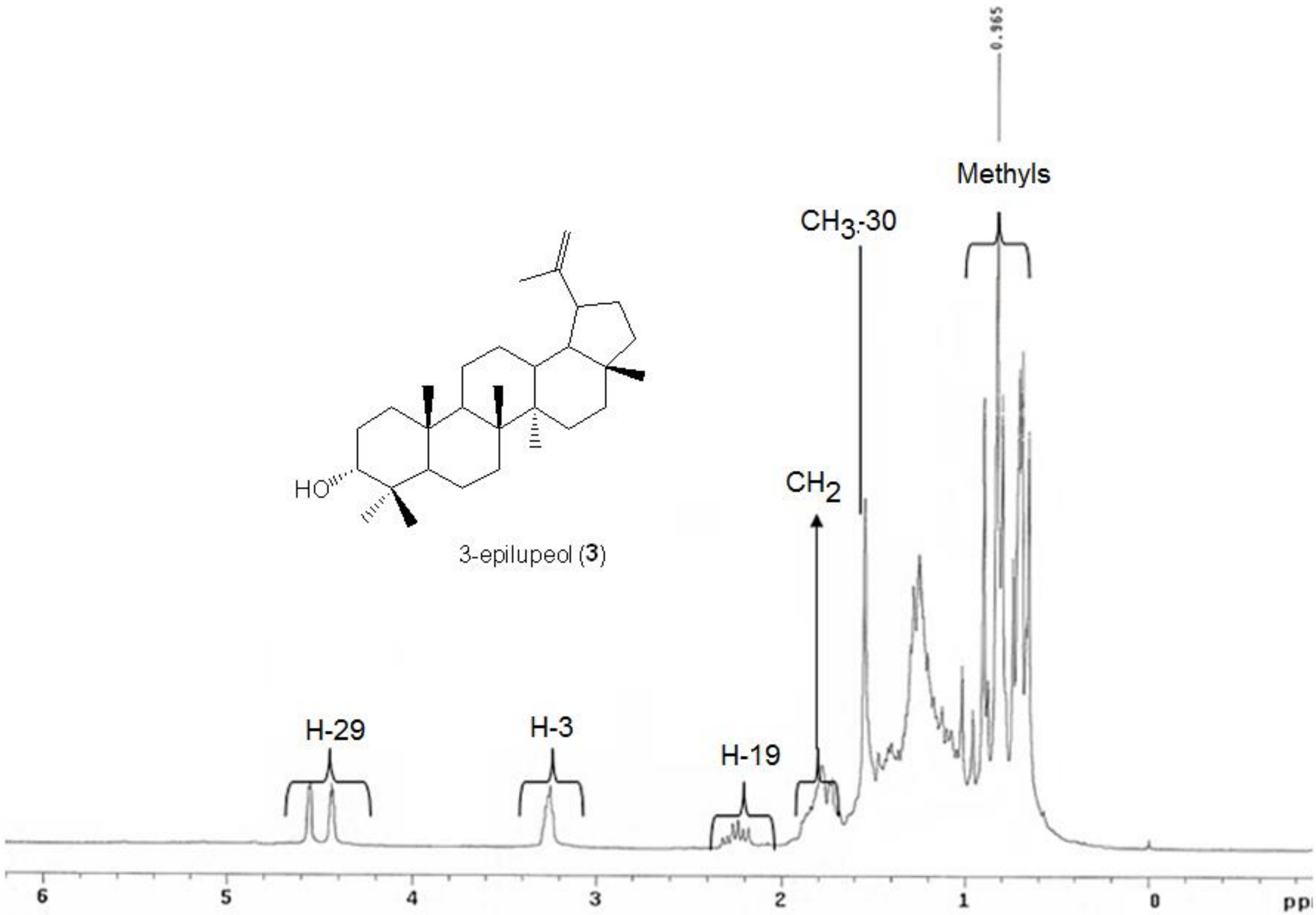

Figure S5. ^{1}H-NMR spectrum (200 MHz, $CDCl_3$) of 3-Epilupeol (3).

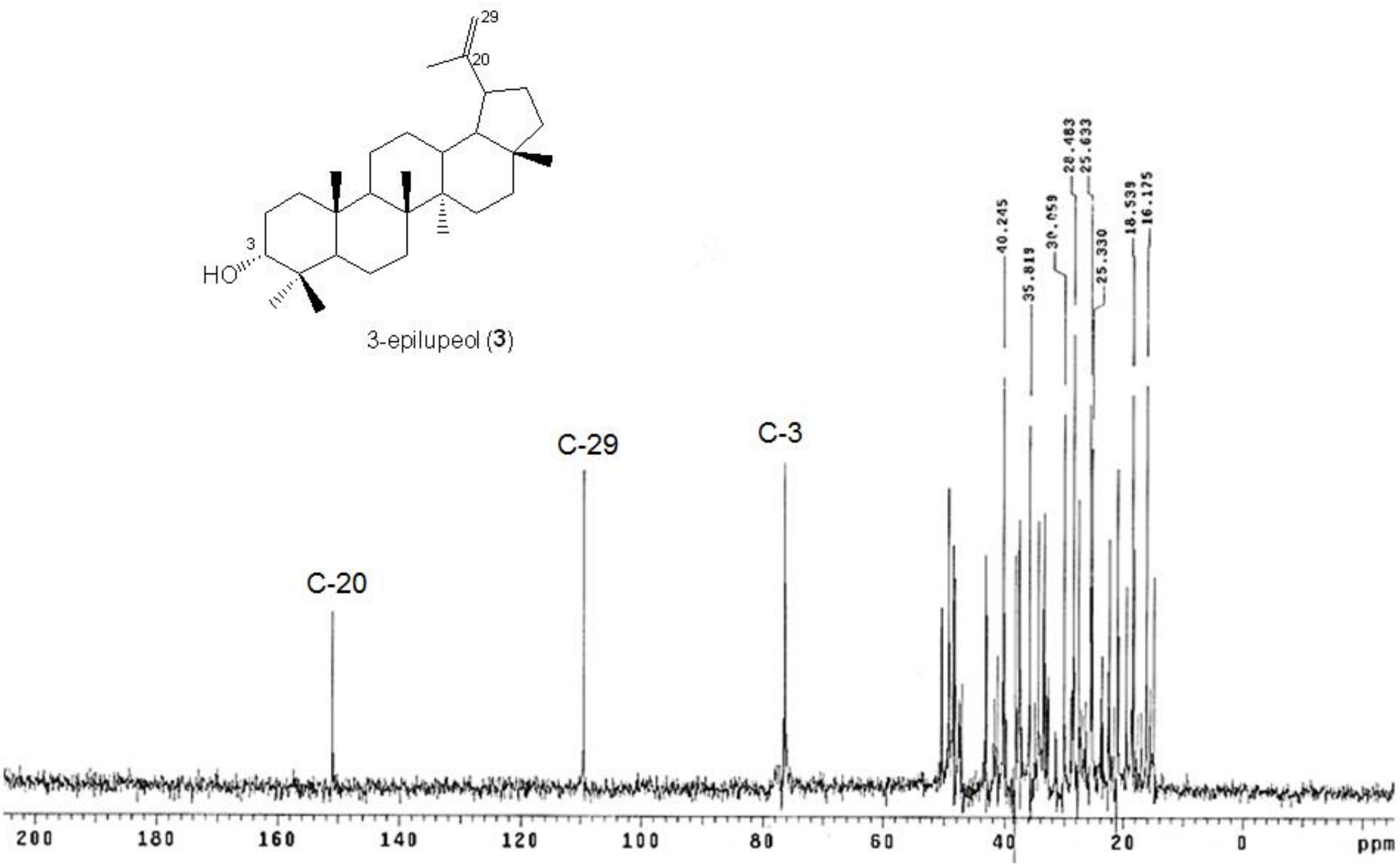

Figure S6. ^{13}C-NMR spectrum (50 MHz, $CDCl_3$) of 3-Epilupeol (3).

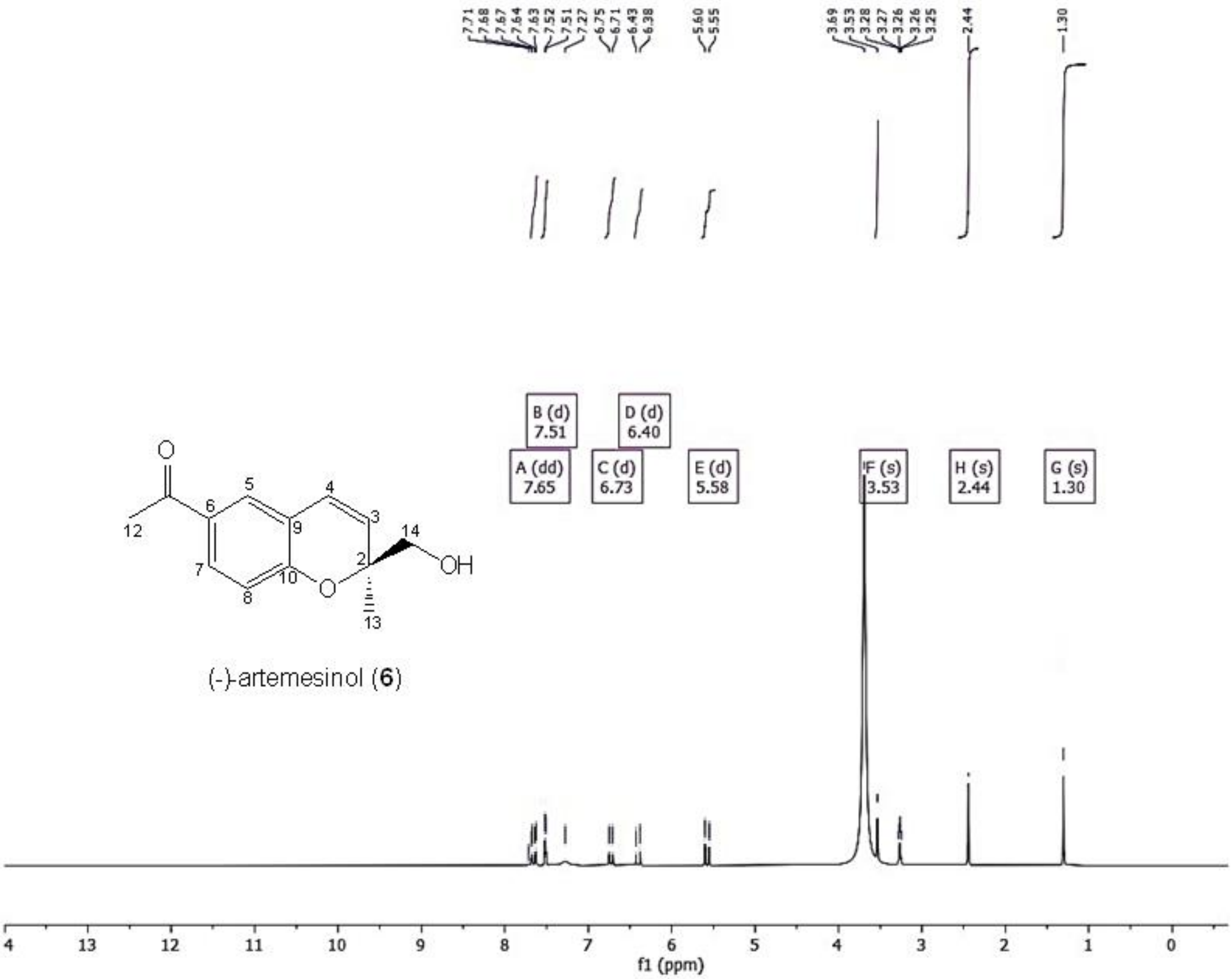

Figure S7. ^{1}H NMR (200 MHz, $CDCl_3$:CD_3OD) of (-)-Artemesinol (6).

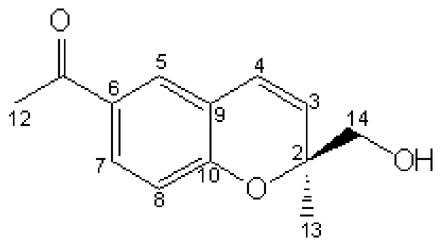

(-)-artemesinol (**6**)

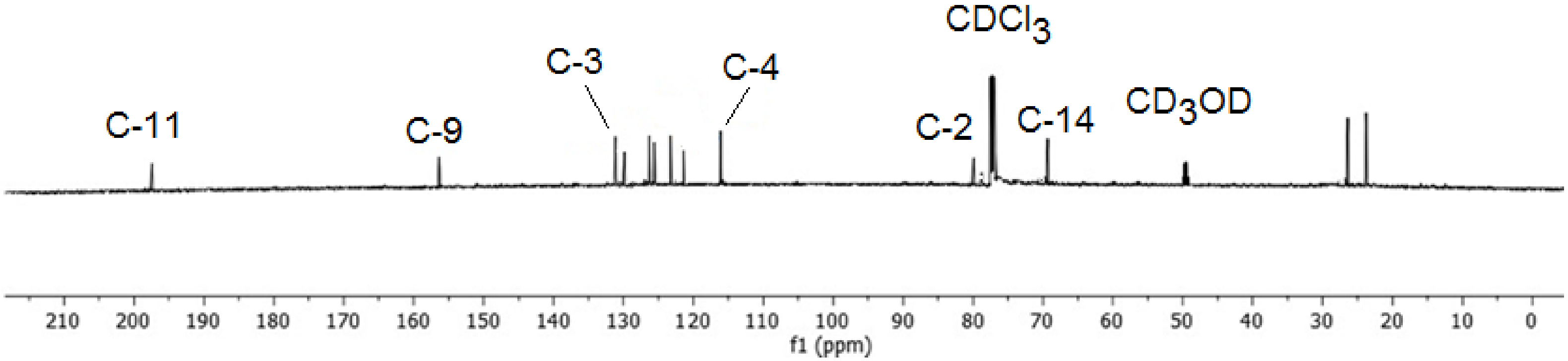

Figure S8. ^{13}C NMR (50 MHz, $CDCl_3$:CD_3OD) of (-)-Artemesinol (6).

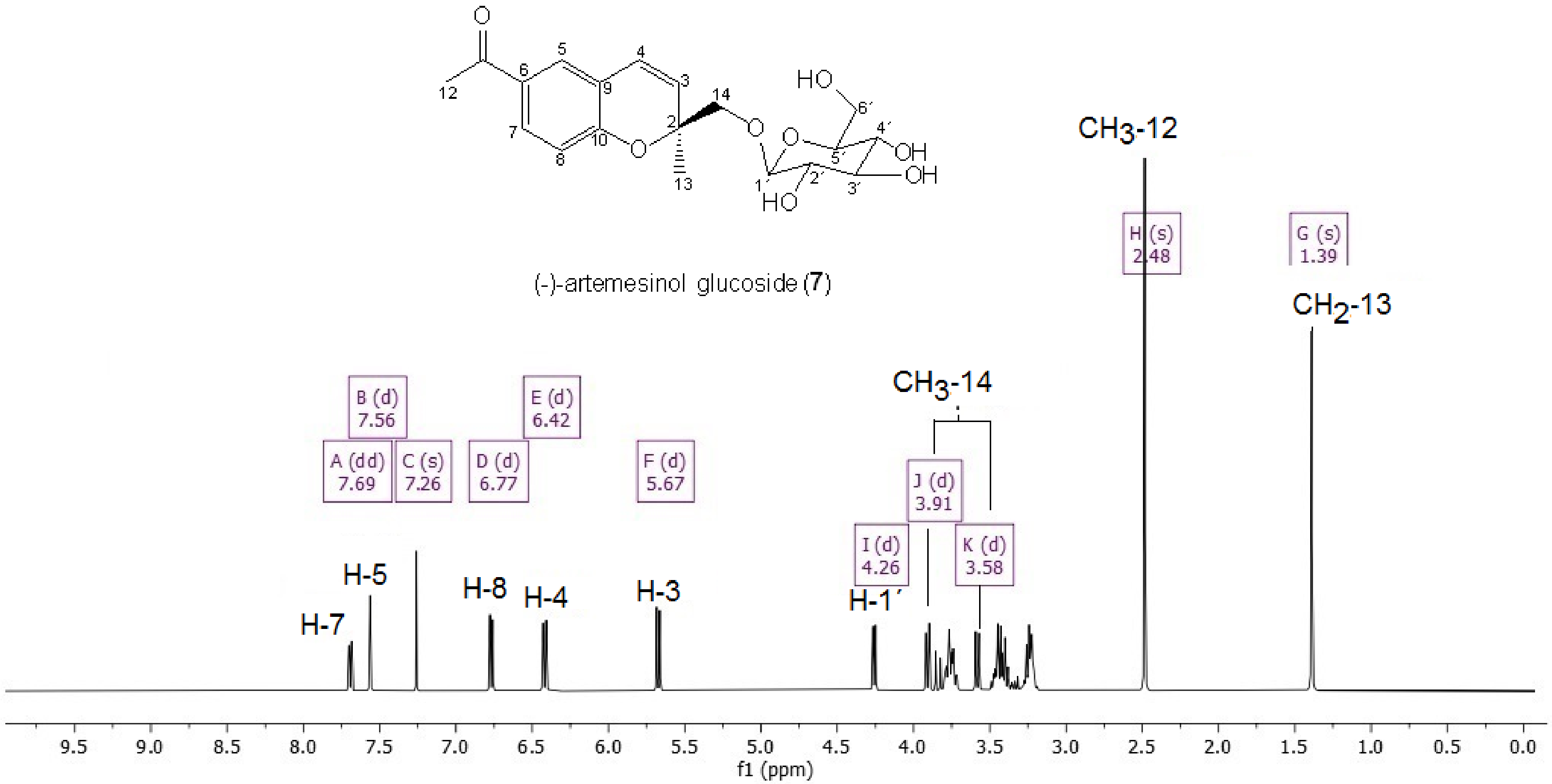

Figure S9. ^{1}H-NMR spectrum (500 MHz, $CDCl_3$: CD_3OD) of (-)-Artemesinol glucoside (7).

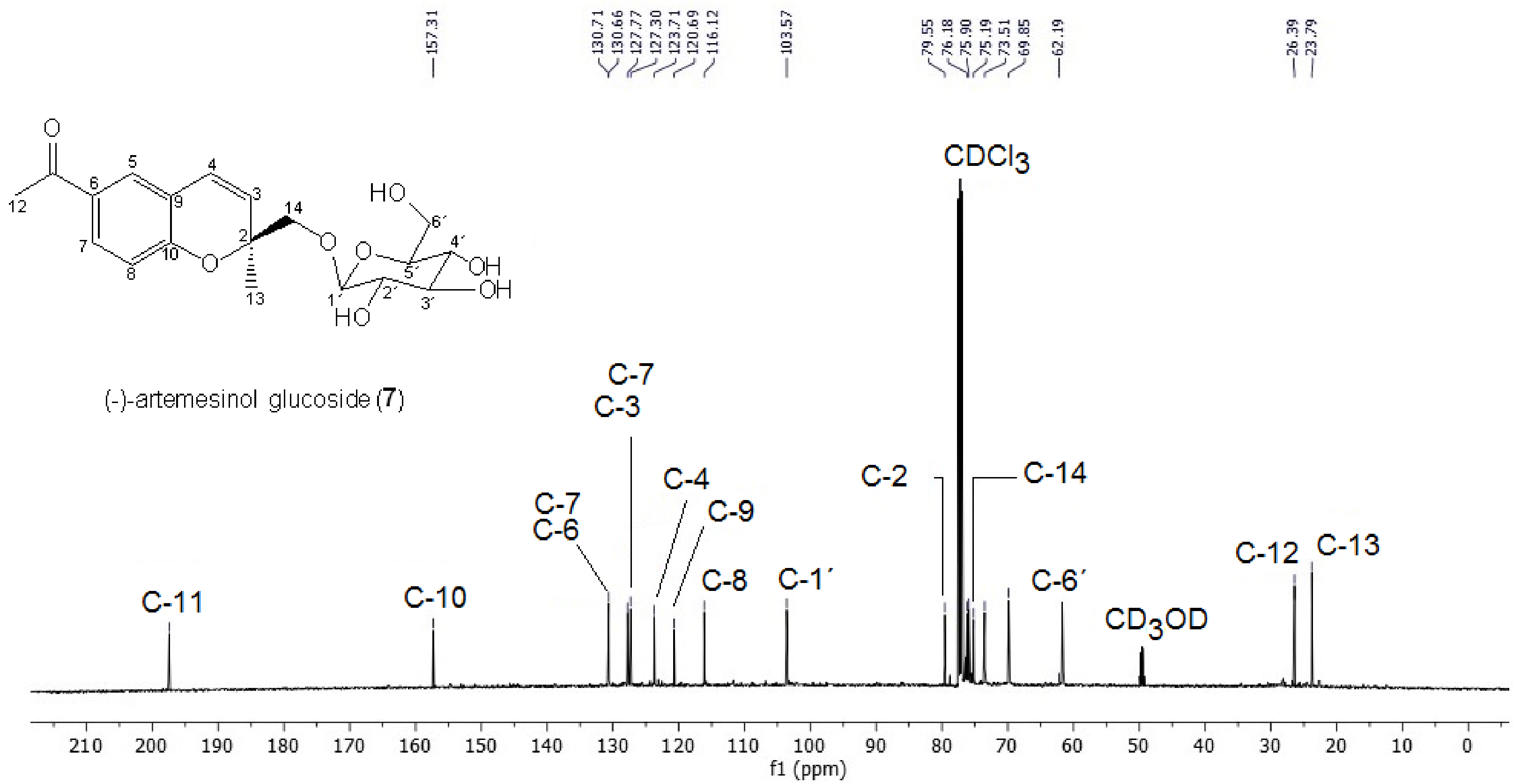

Figure S10. ^{13}C-NMR spectrum (125 MHz, $CDCl_3$: CD_3OD) of (-)-Artemesinol glucoside (7).

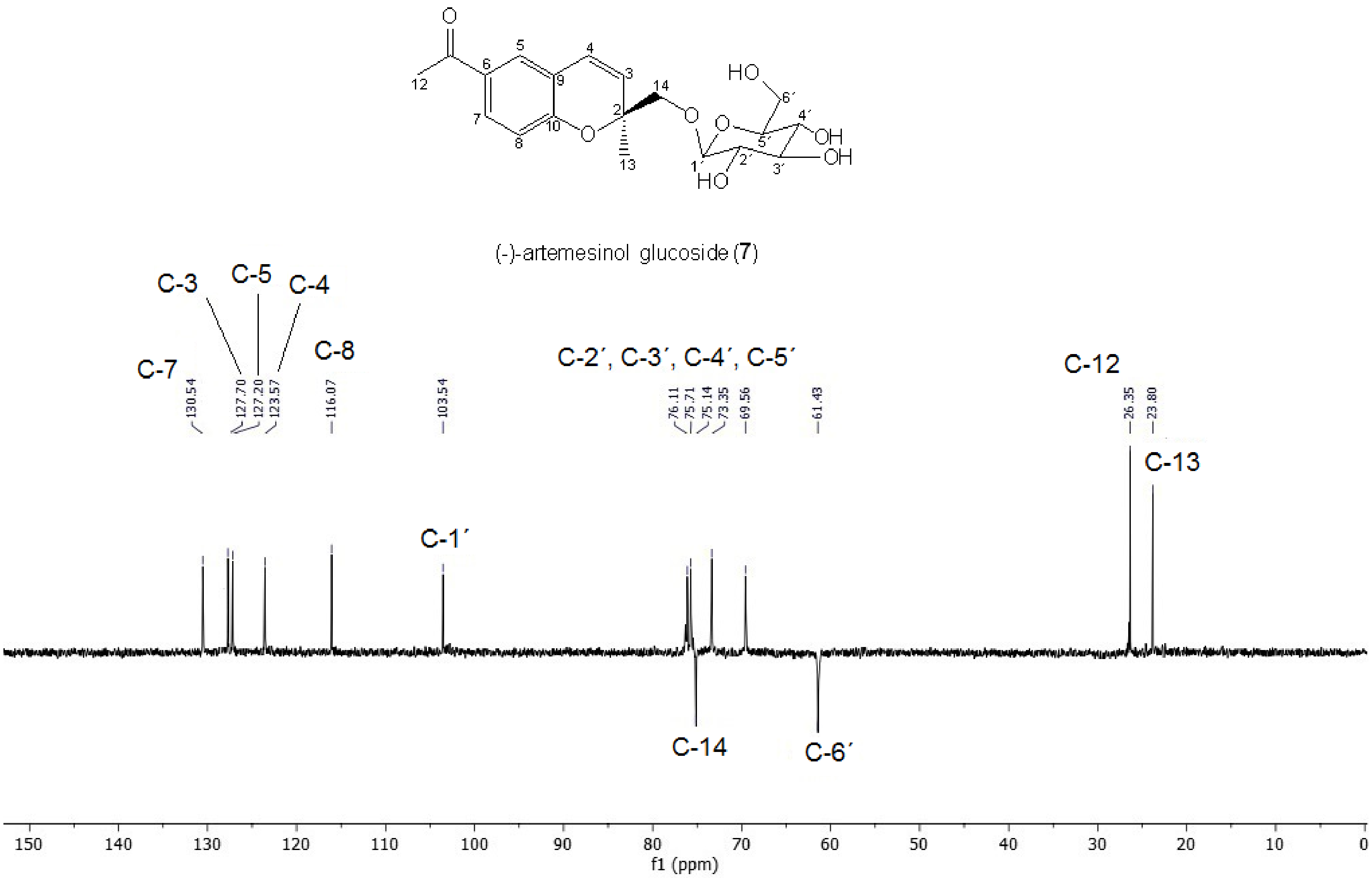

Figure S11. DEPT spectrum (125 MHz, $CDCl_3$: CD_3OD) of (-)-Artemesinol glucoside (7).

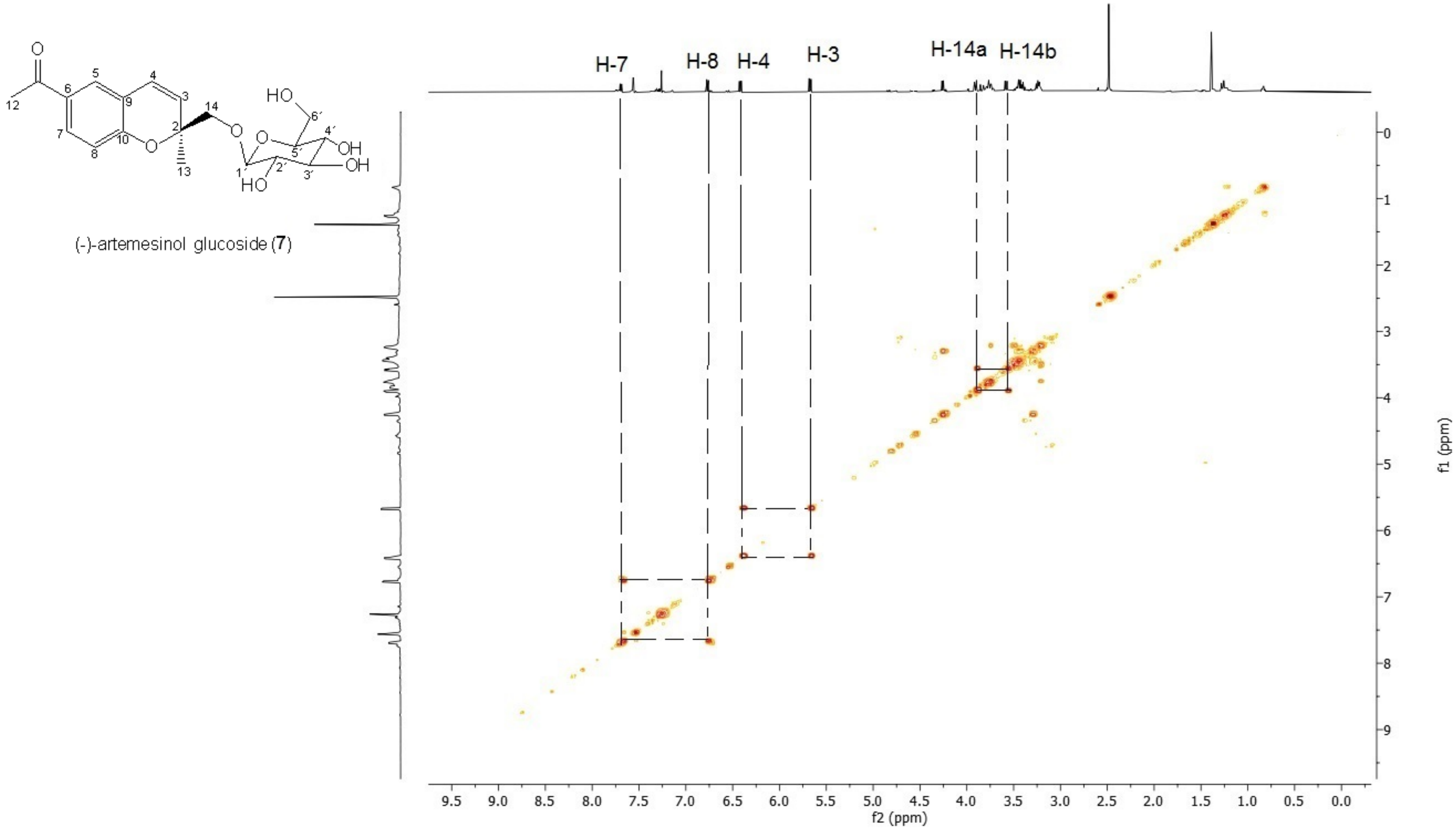

Figure S12. COSY spectrum (500 MHz, $CDCl_3$: CD_3OD) of (-)-Artemesinol glucoside (7).

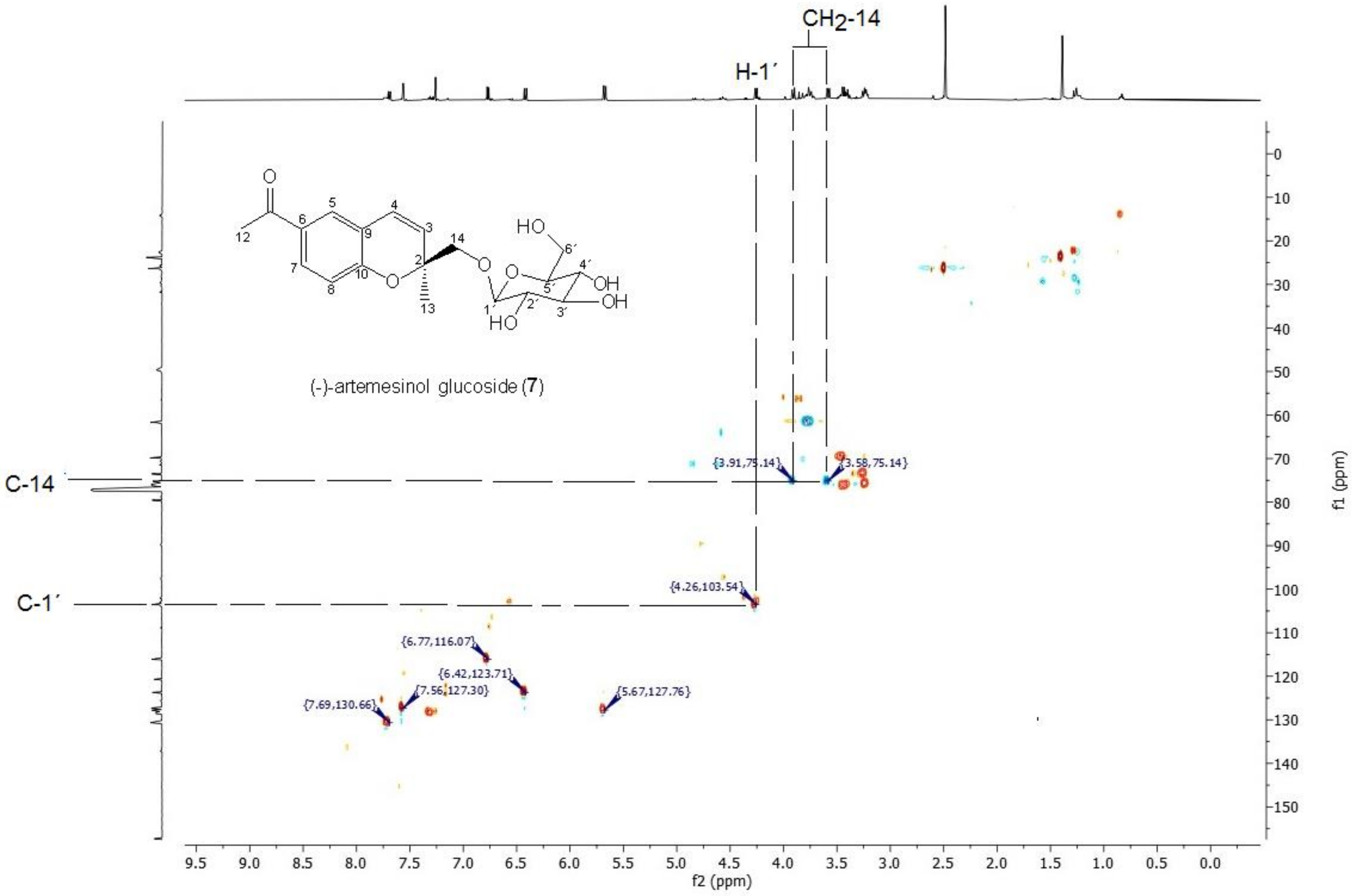

Figure S13. HSQC spectrum (500 MHz, $CDCl_3$: CD_3OD) of (-)-Artemesinol glucoside (7).

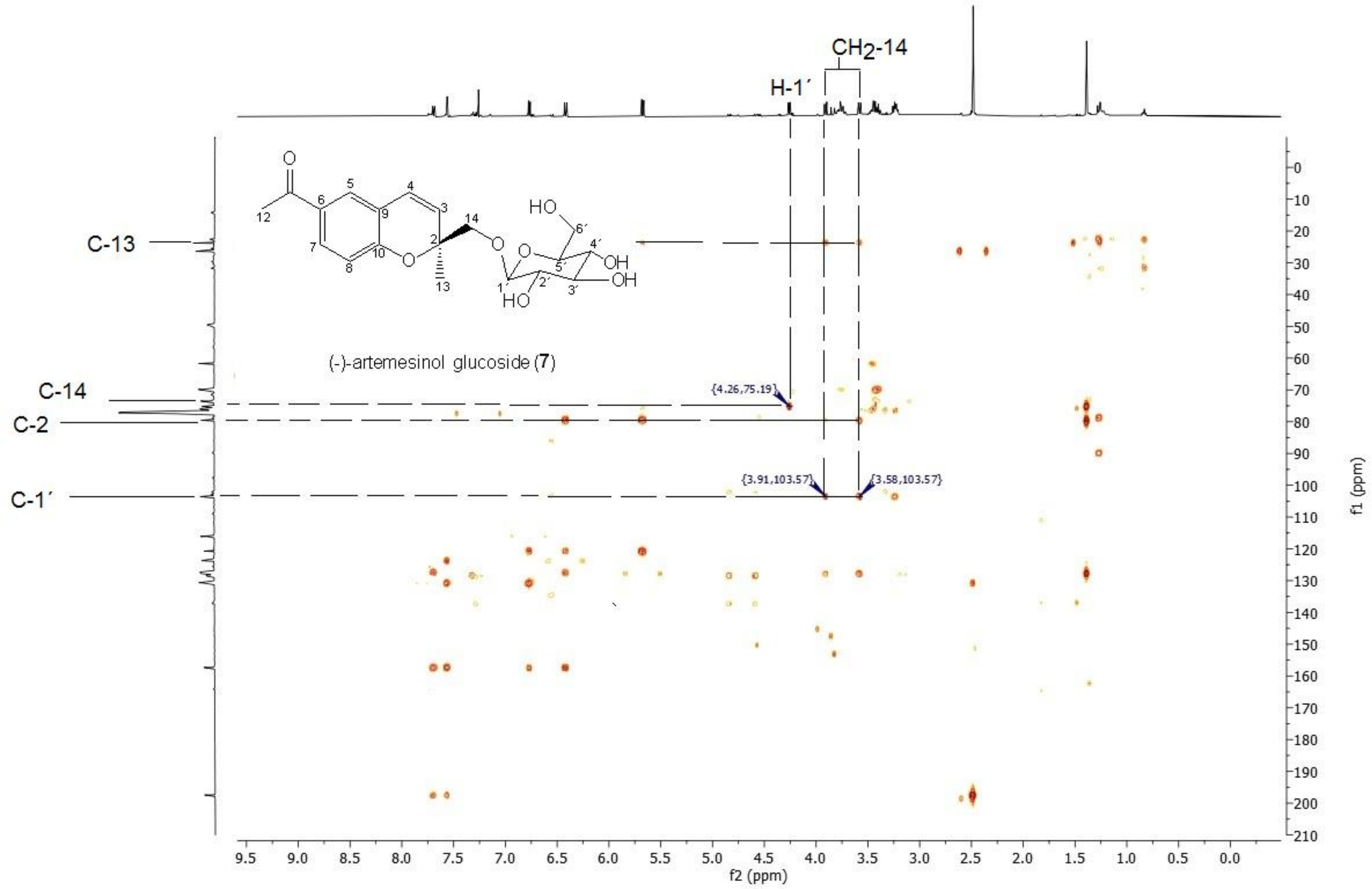

Figure S14. HMBC spectrum (500 MHz, $CDCl_3$: CD_3OD) of (-)-Artemesinol glucoside (7).

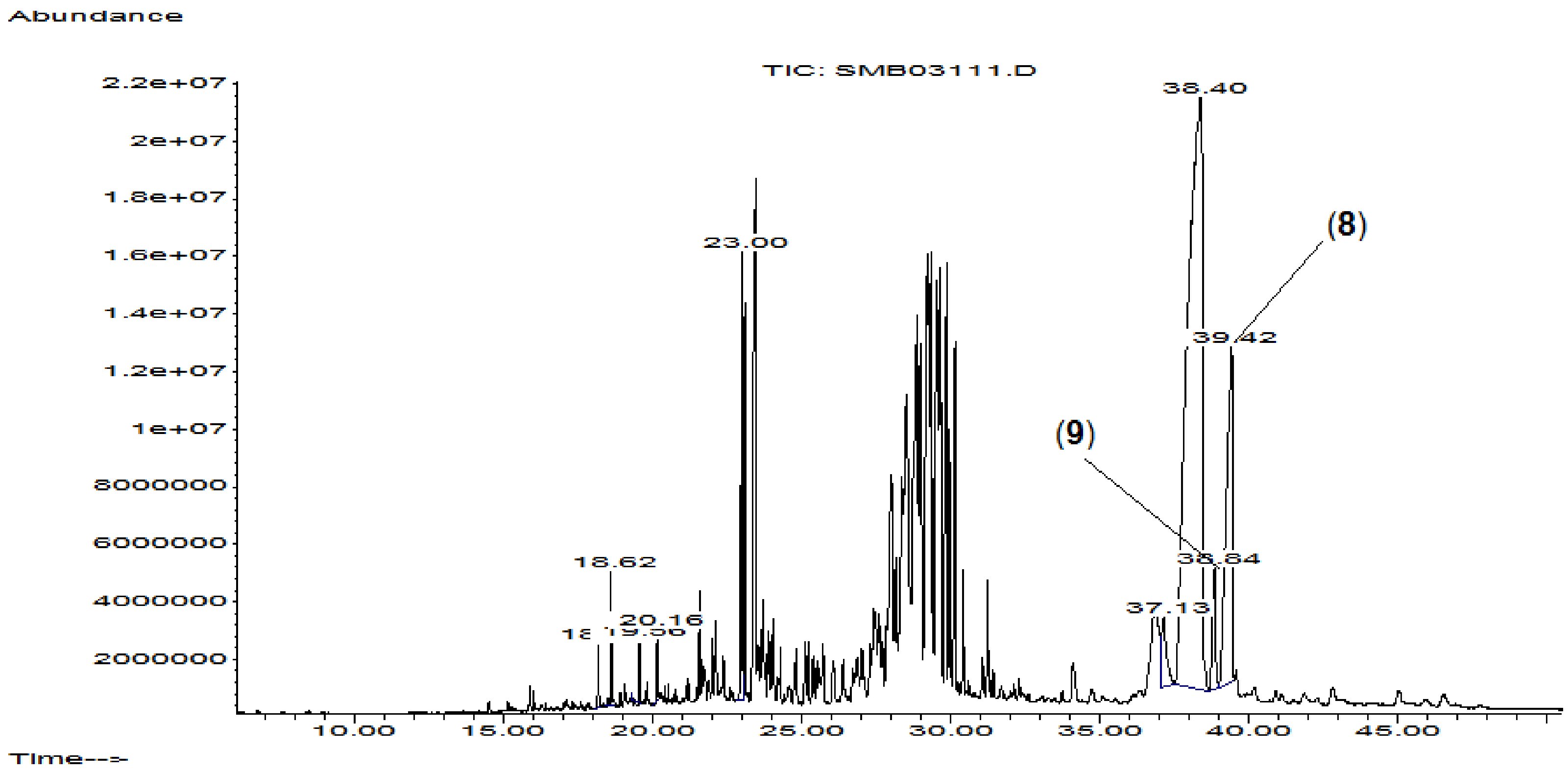

Figure S15a. GC-MS chromatogram of cell suspension culture extract with compounds 8 and 9.

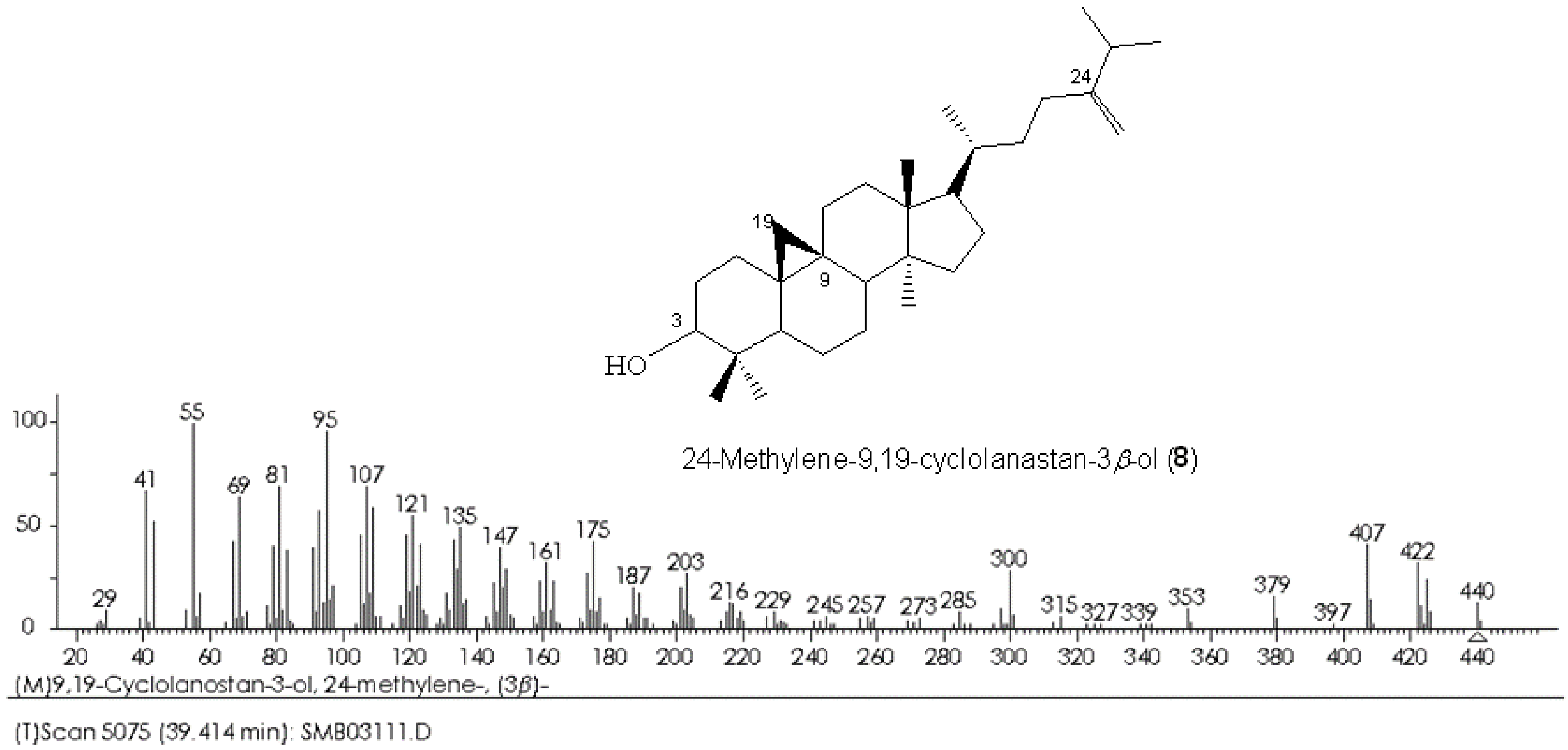

Figure S15b. Mass spectrum of compound 8.

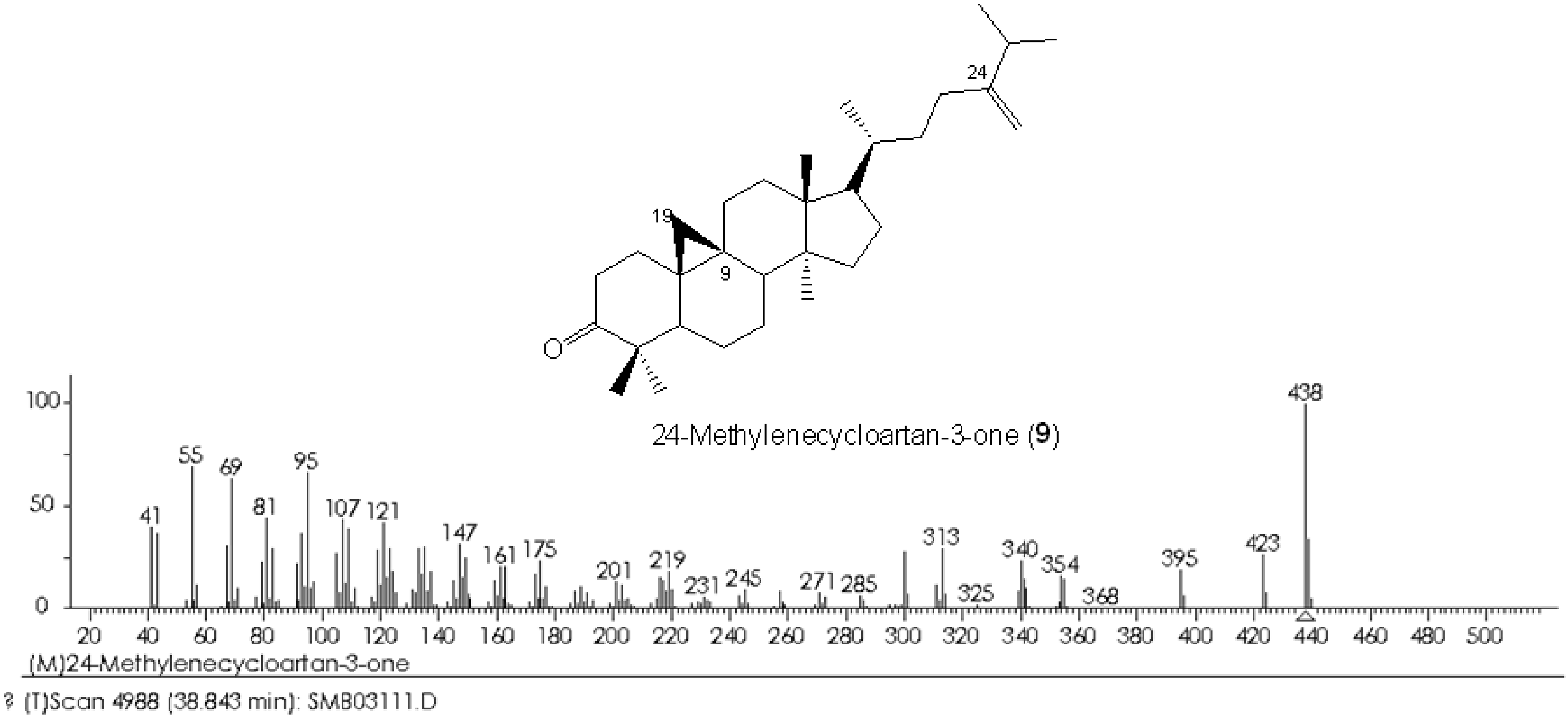

Figure S15c. Mass spectrum of compound 9.

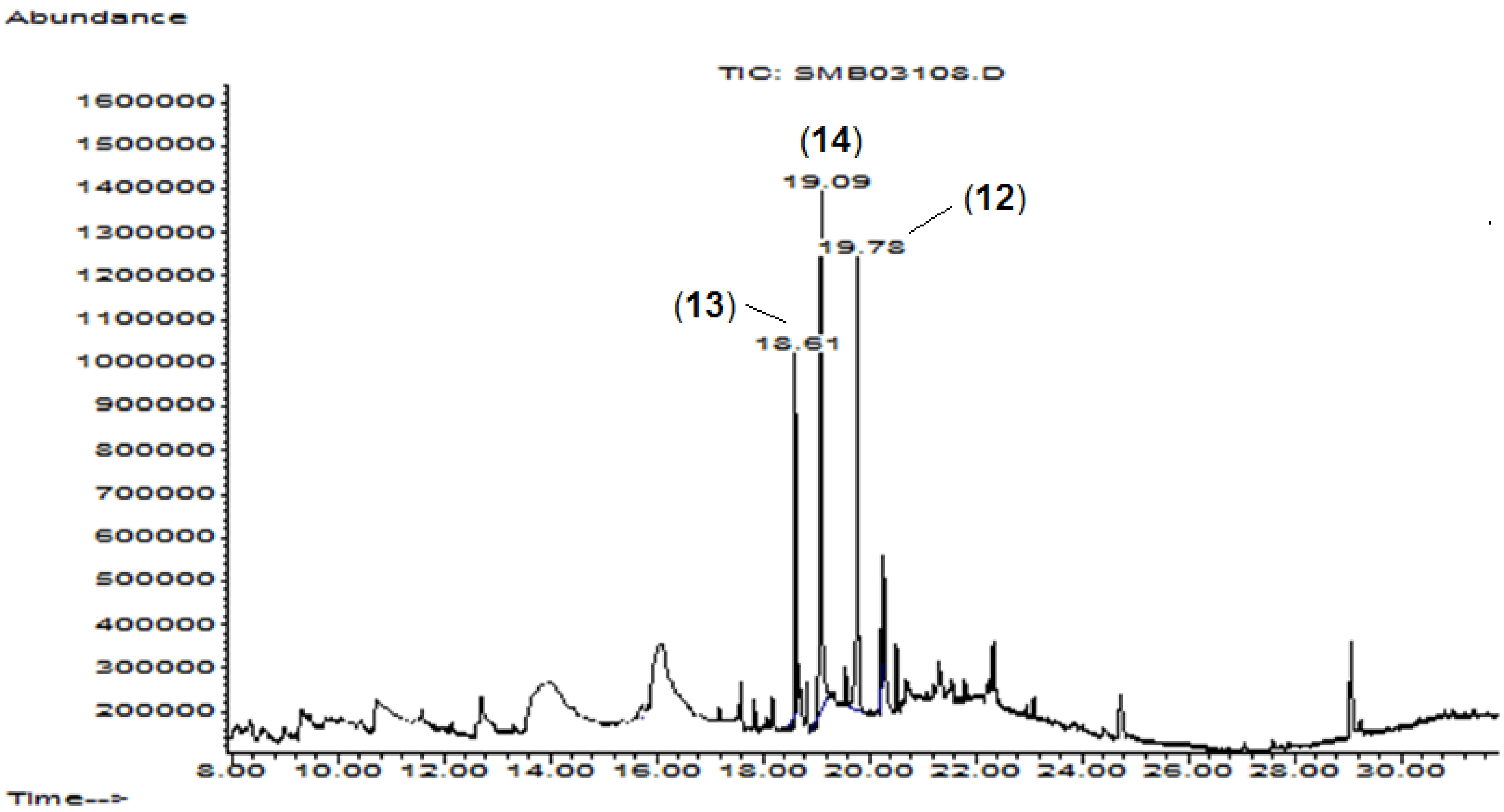

Figure S16a. GC-MS chromatogram of cell suspension culture extract with compounds 12–14.

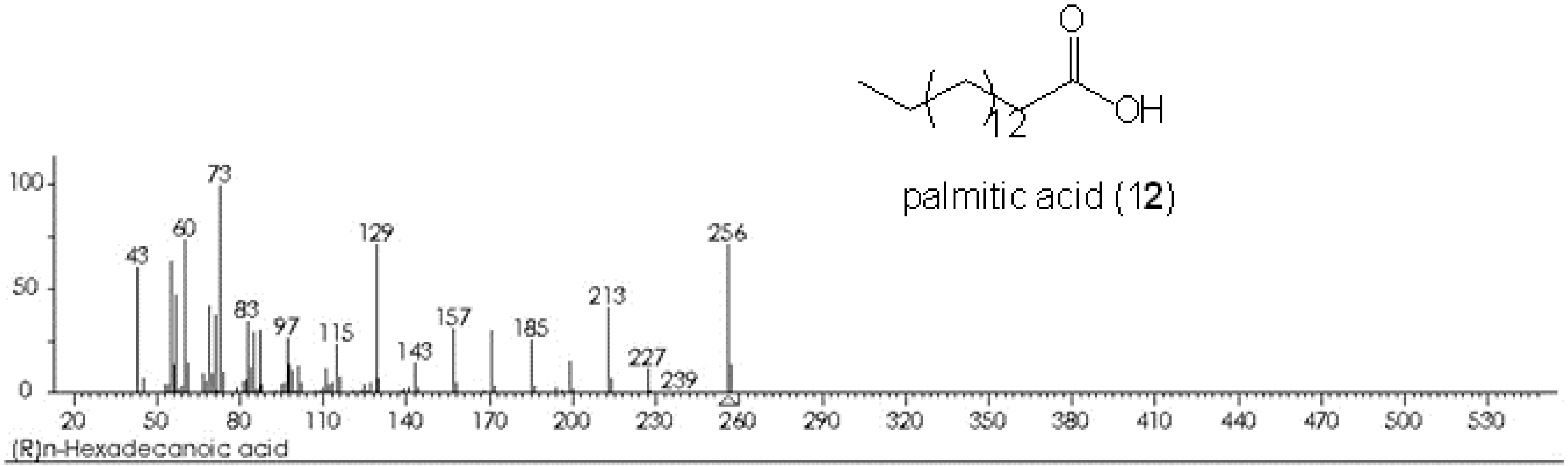

Figure S16b. Mass spectrum of compound 12.

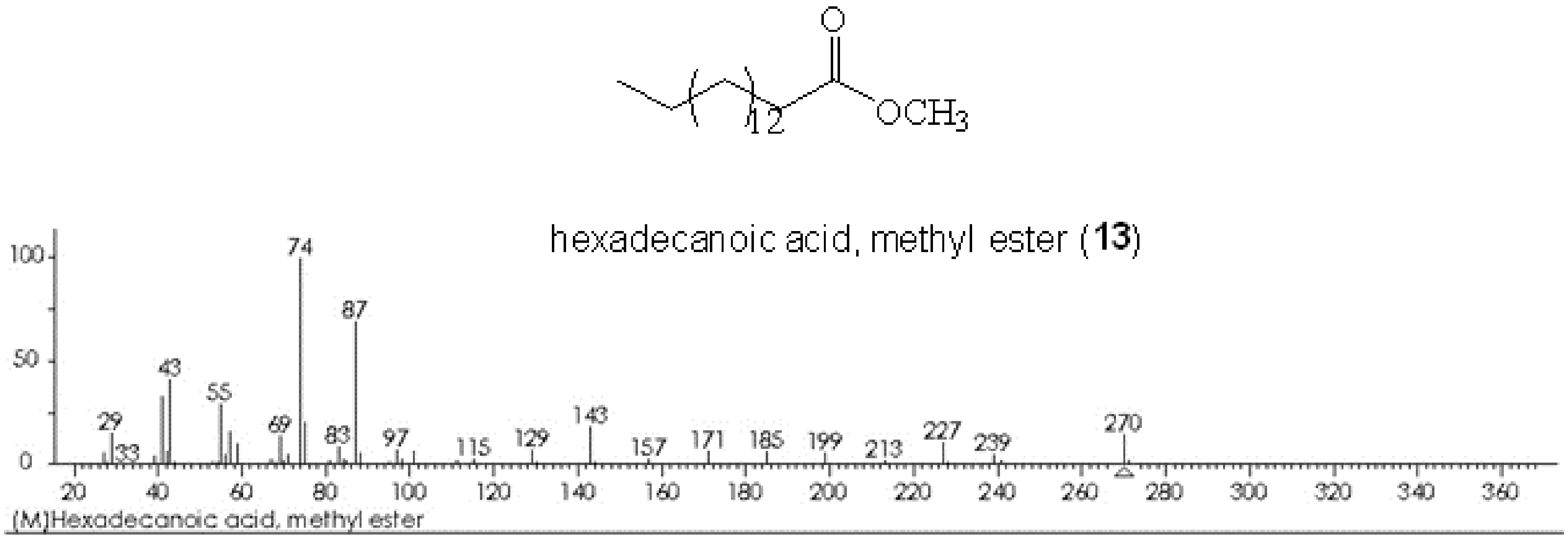

Figure S16c. Mass spectrum of compound 13.

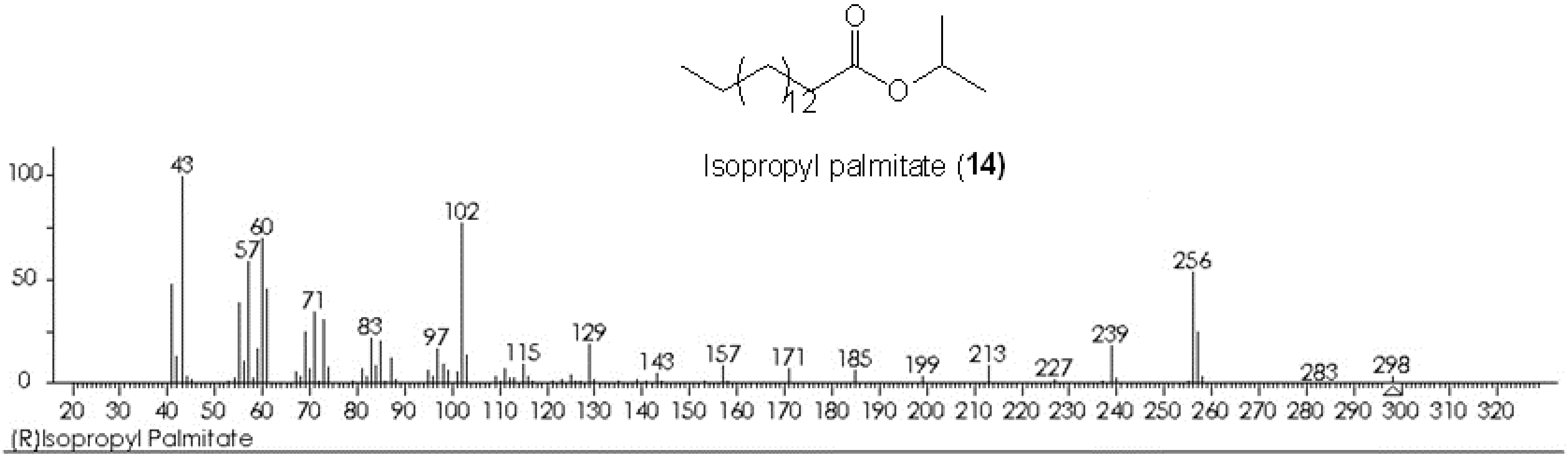

Figure S16d. Mass spectrum of compound 14.

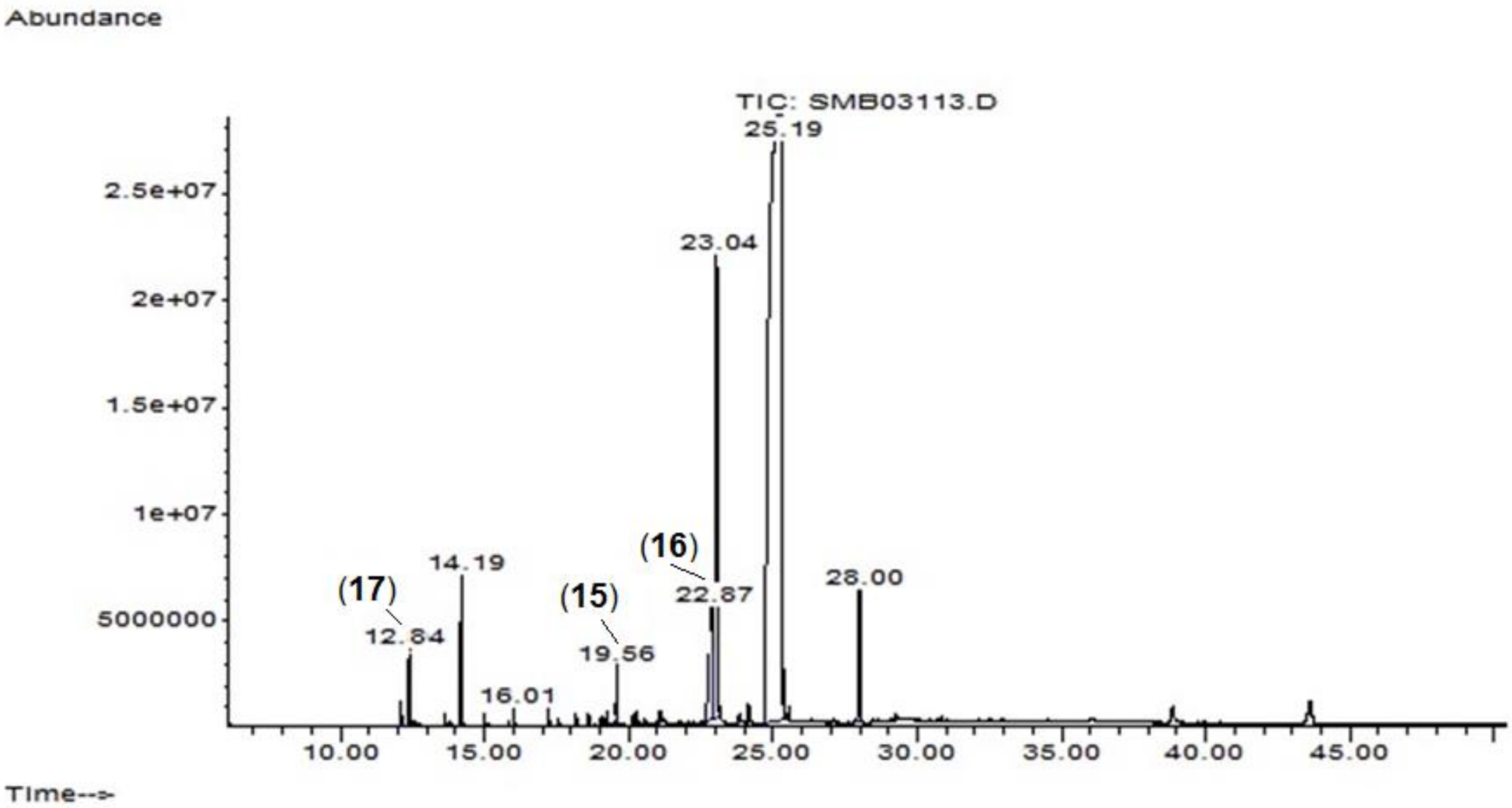

Figure S17a. GC-MS chromatogram of cell suspension culture extract with compounds 15–17.

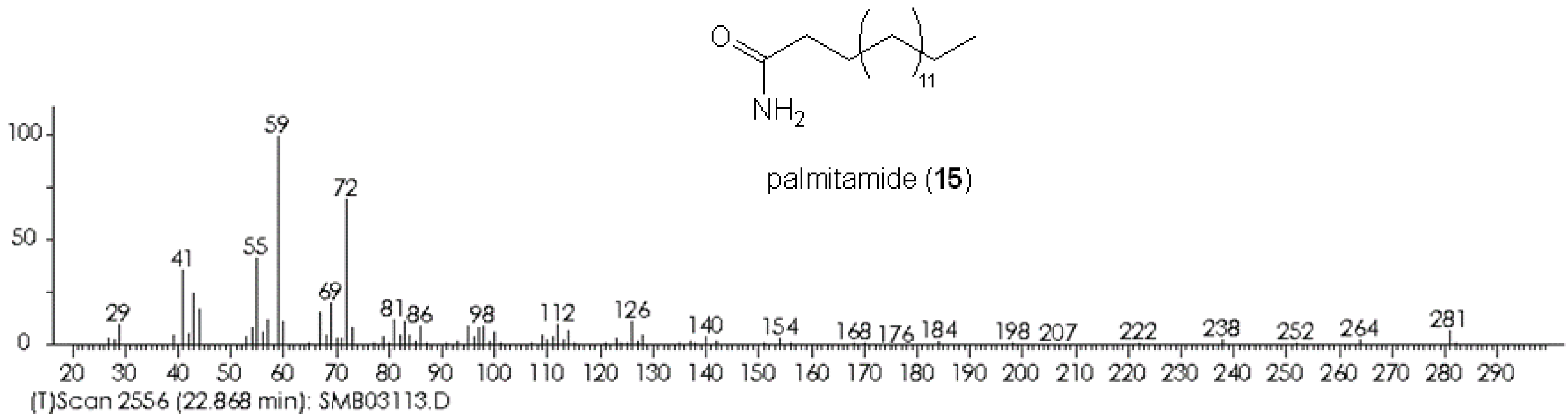

Figure S17b. Mass spectrum of compound 15.

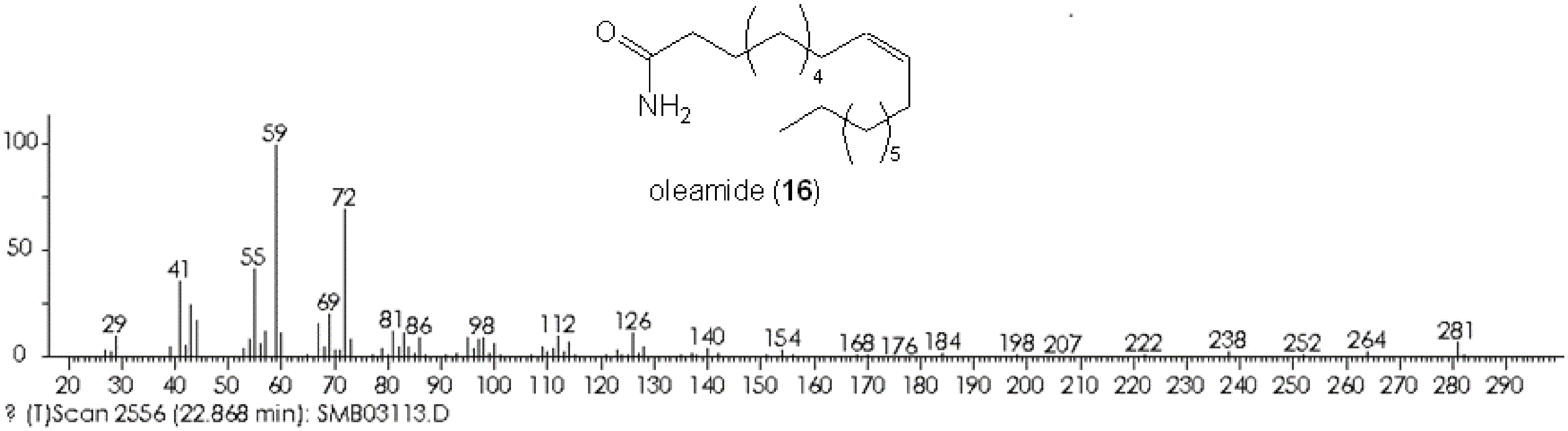

Figure S17c. Mass spectrum of compound 16.

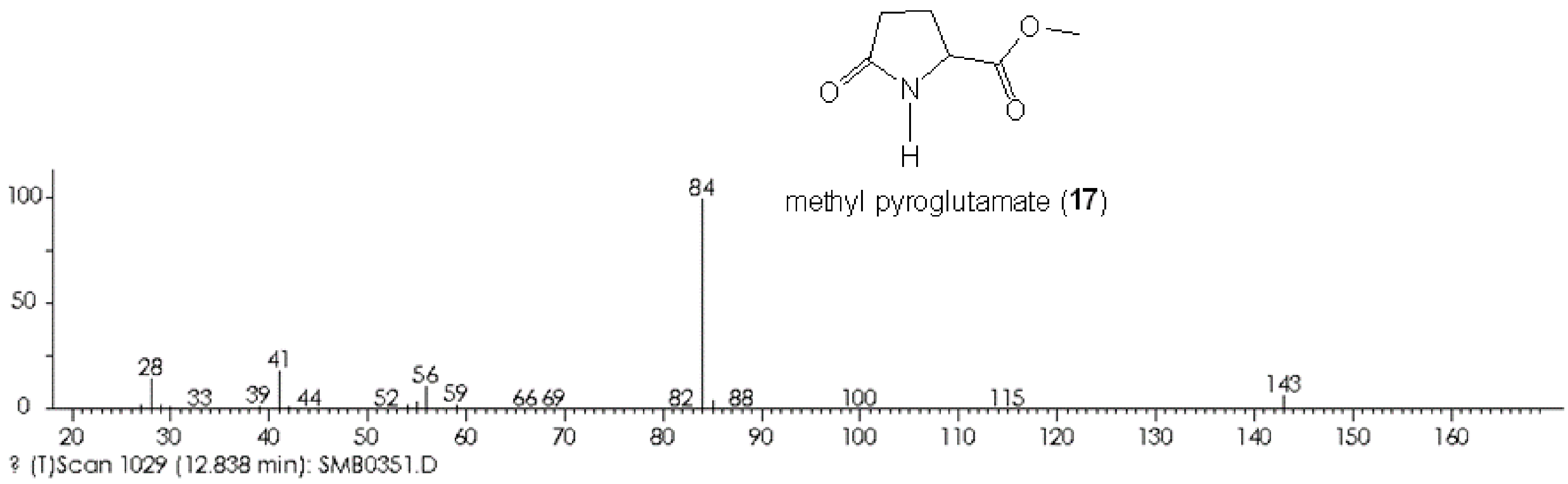

Figure S17d. Mass spectrum of compound 17.

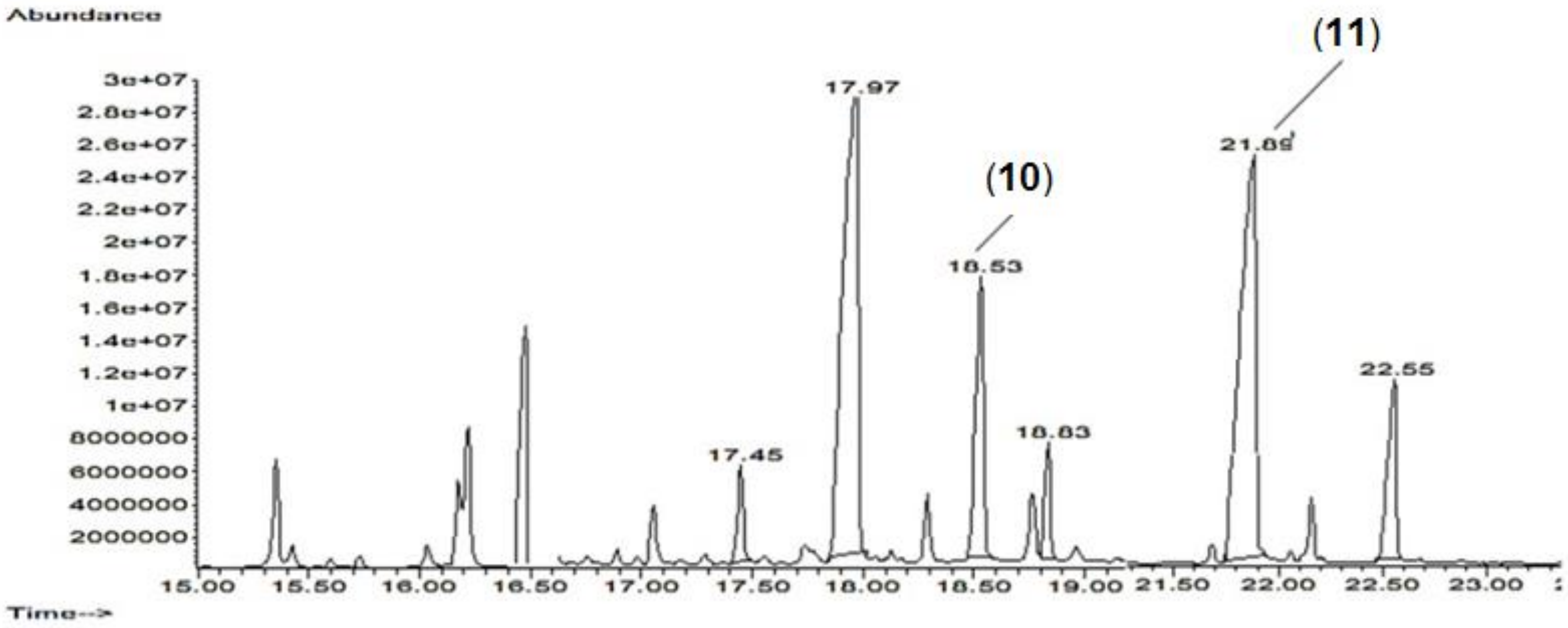

Figure S18a. GC-MS chromatogram of compounds 10–11 obtained of the ethyl acetate extract from wild plant *A. pichinchensis*.

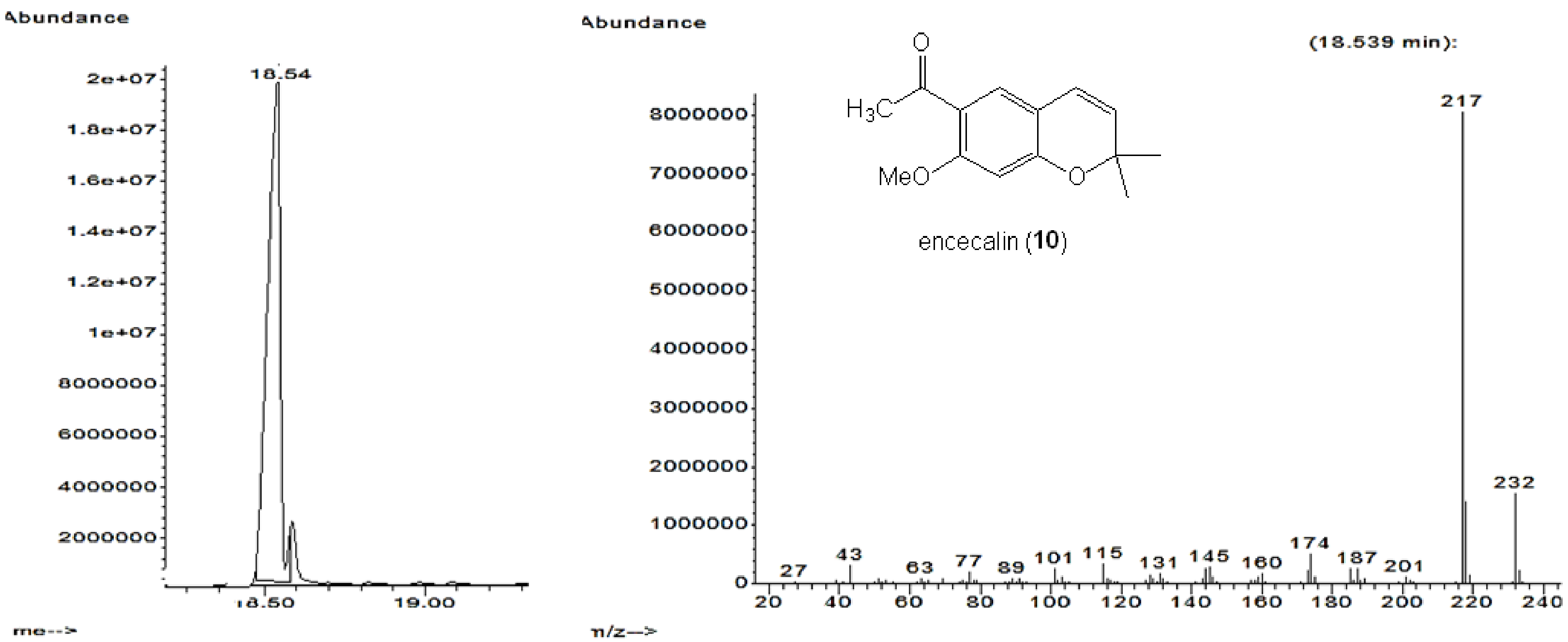

Figure S18b. GC-MS chromatograms of compound 10.

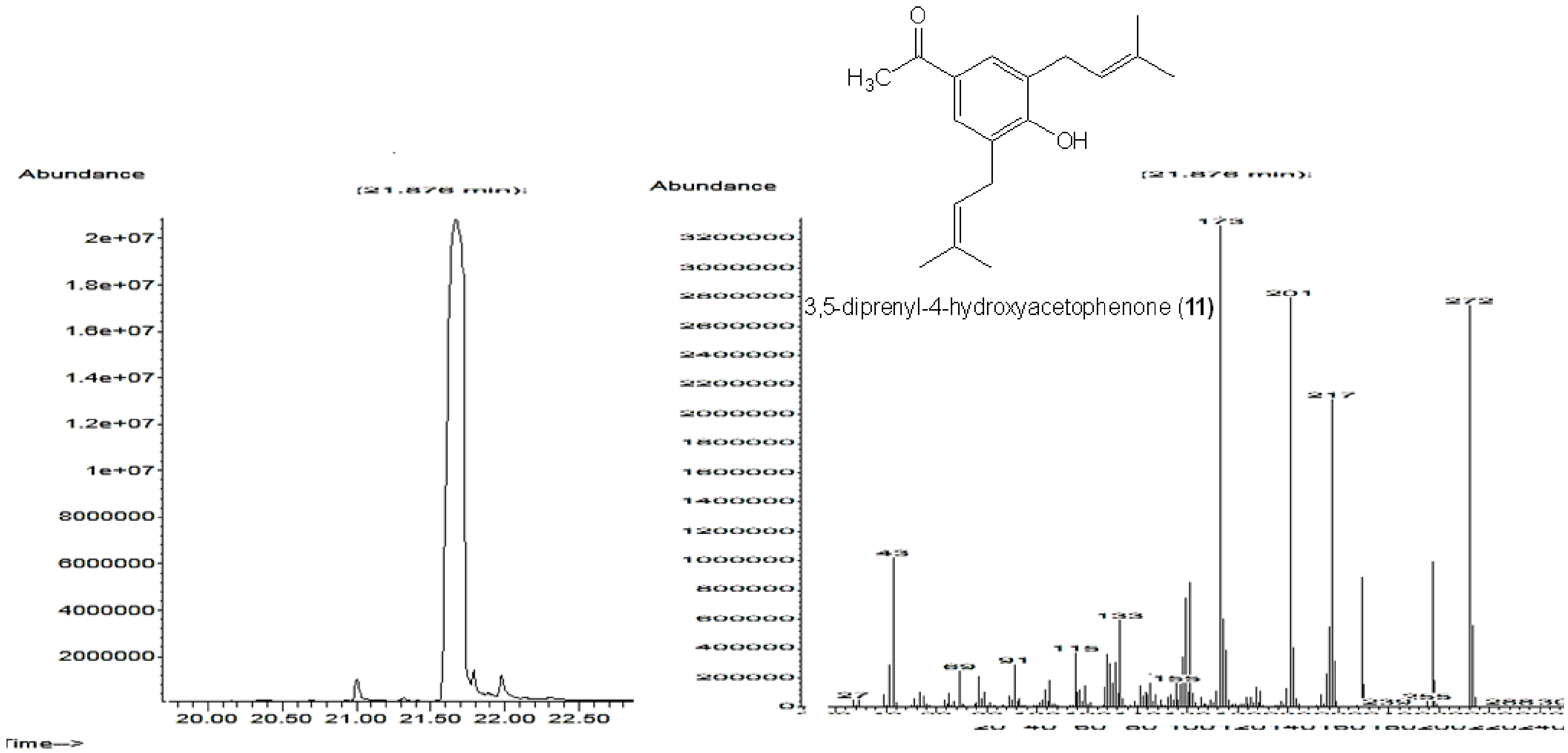

Figure S18c. GC-MS chromatograms of compound 11.

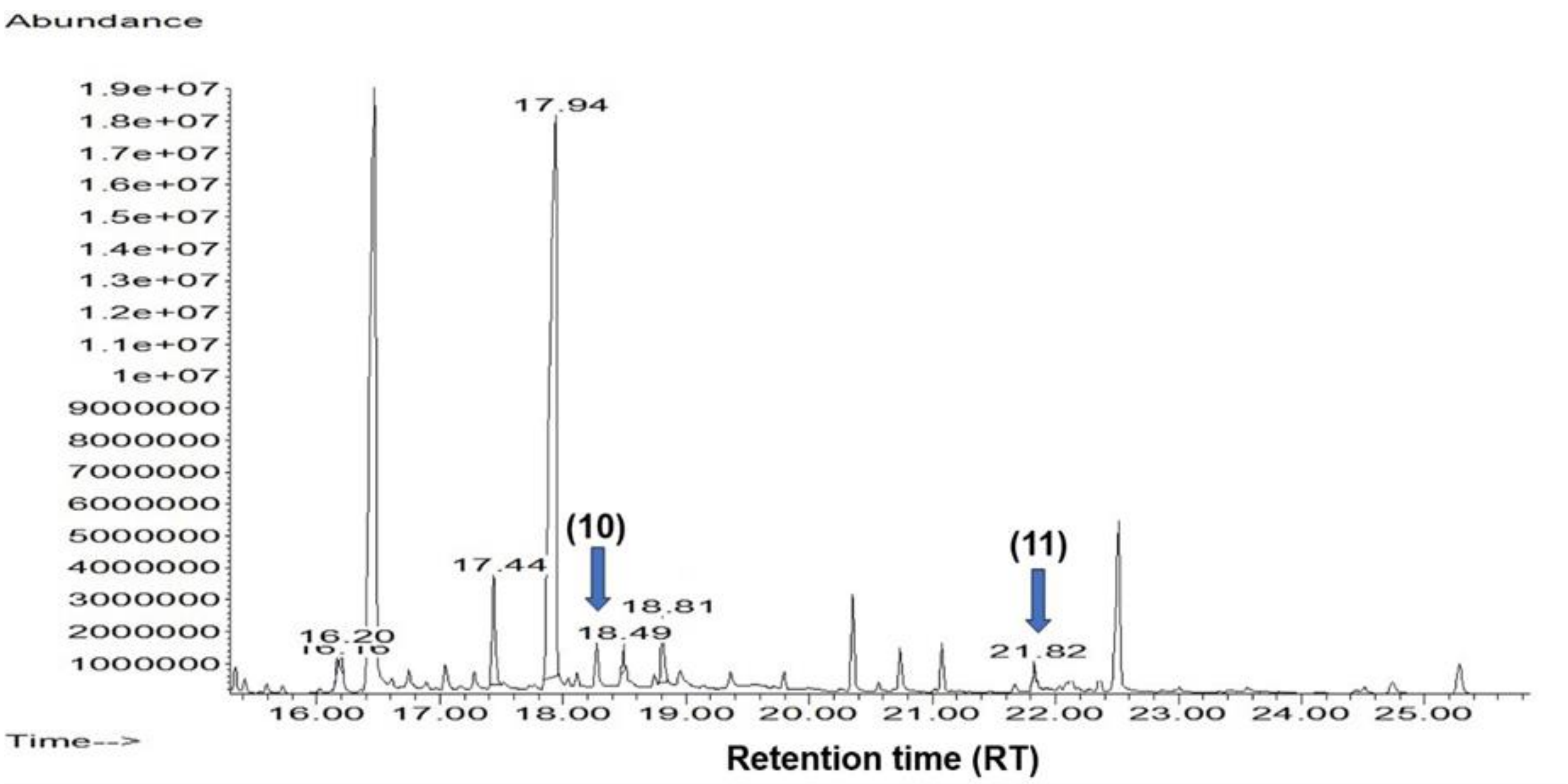

Figure S18d. GC-MS chromatograms of the compounds encecalin (10) and 3,5-diprenyl-4-hydroxyacetophenone (11) obtained of the ethyl acetate extract from *A. pichinchensis* cell suspension cultures.

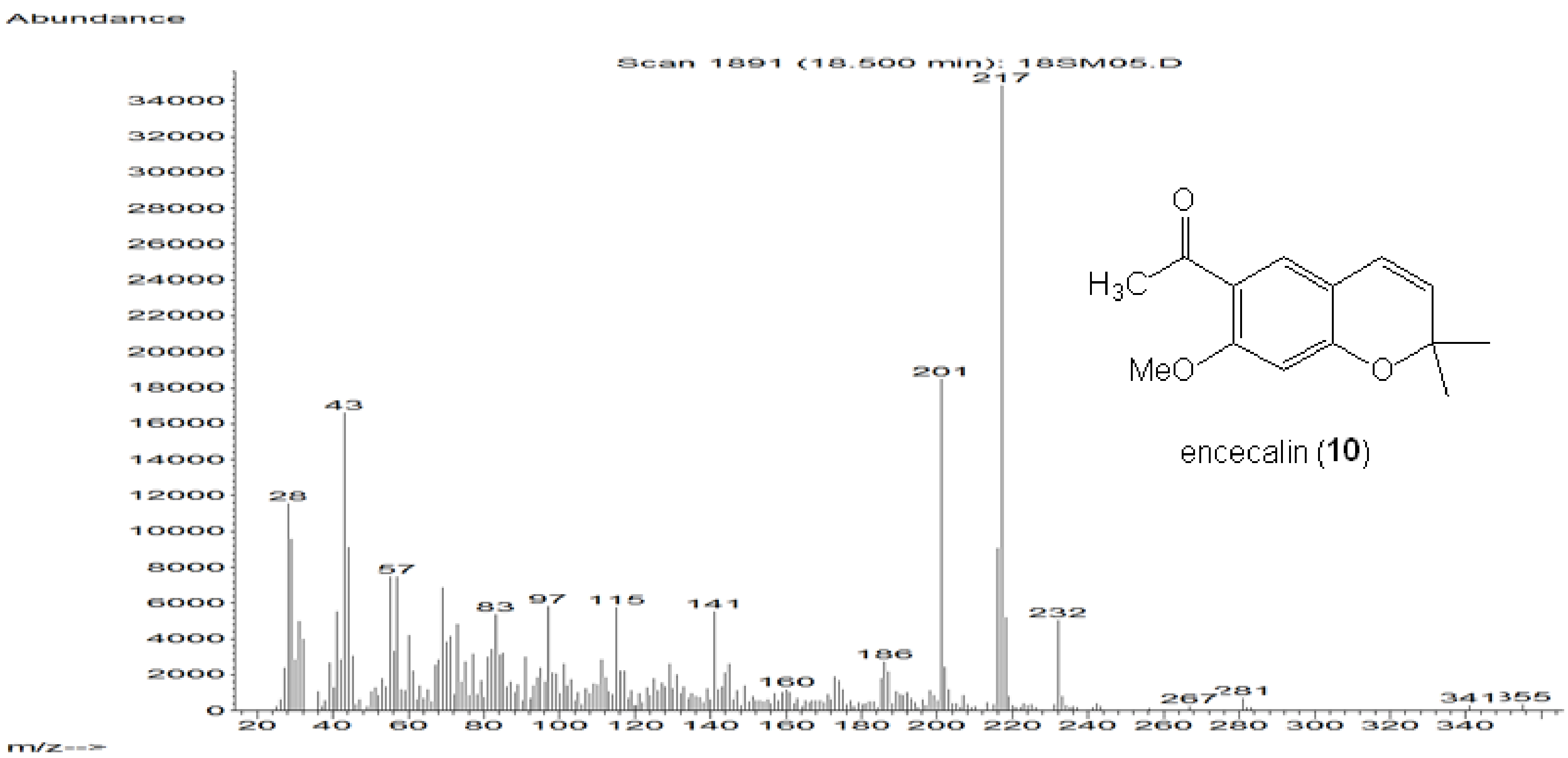

Figure S18e. GC-MS chromatogram of compound 10 obtained from acetate ethyl extract of cell suspension cultures.

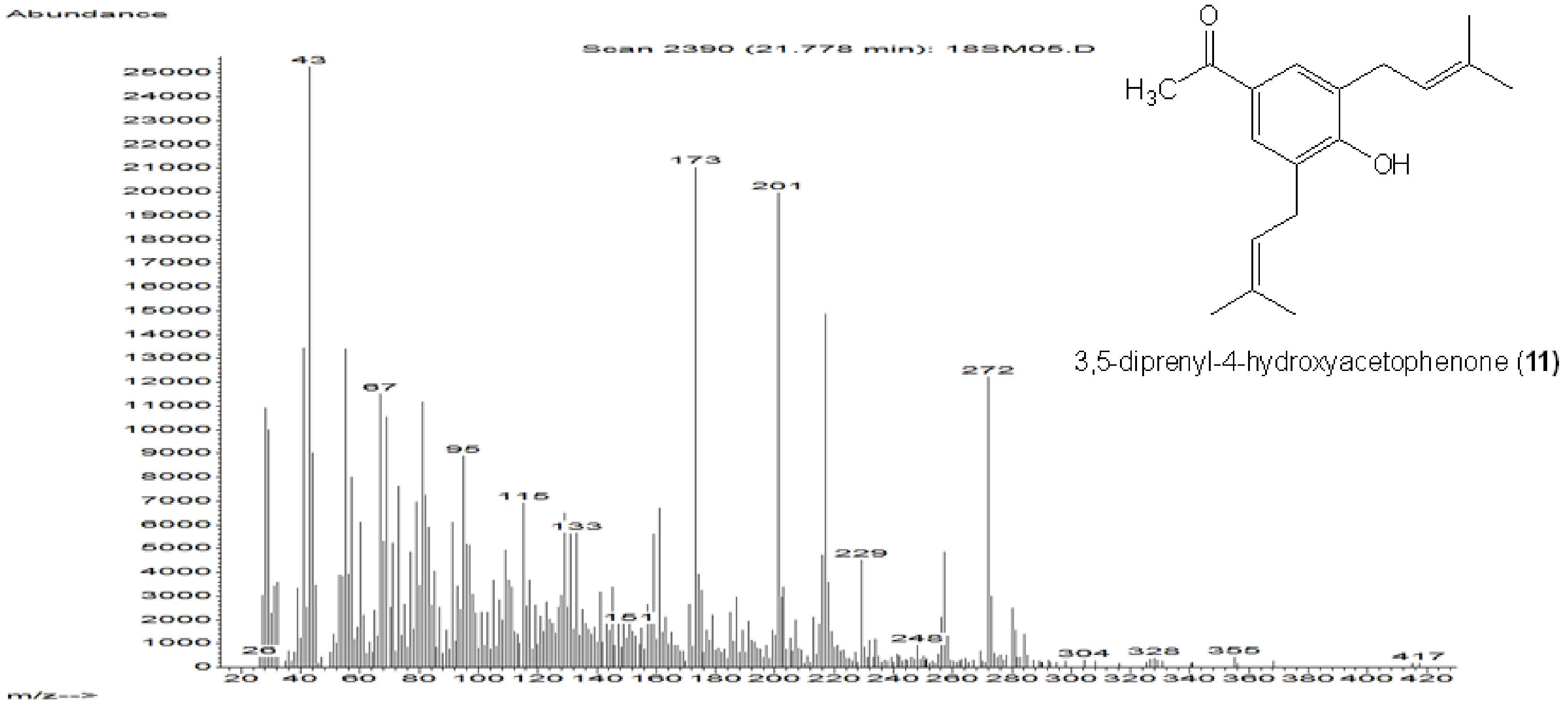

Figure S18f. GC-MS chromatogram of compound 11 obtained from acetate ethyl extract of cell suspension cultures.

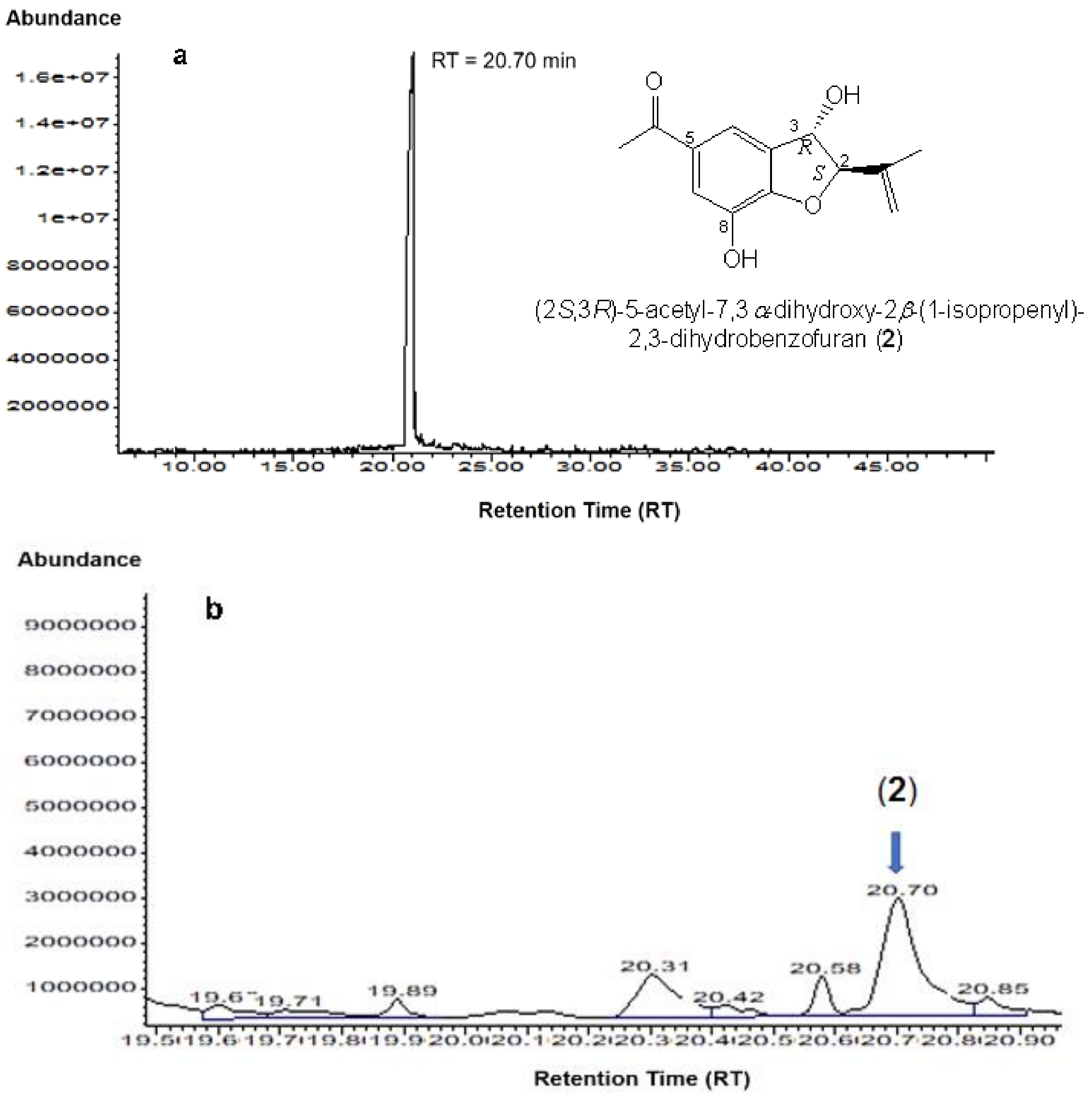

Figure S19. GC-MS chromatograms of standard compound and EtOAc extract. (**a**) 2,3-dihydrobenzofuran (**2**) profile used as a standard; (**b**) EtOAc extract profile from *A. pichinchensis* cell suspension culture at 8 days of culture showing the peak of 2,3-dihydrobenzofuran (**2**) compound.

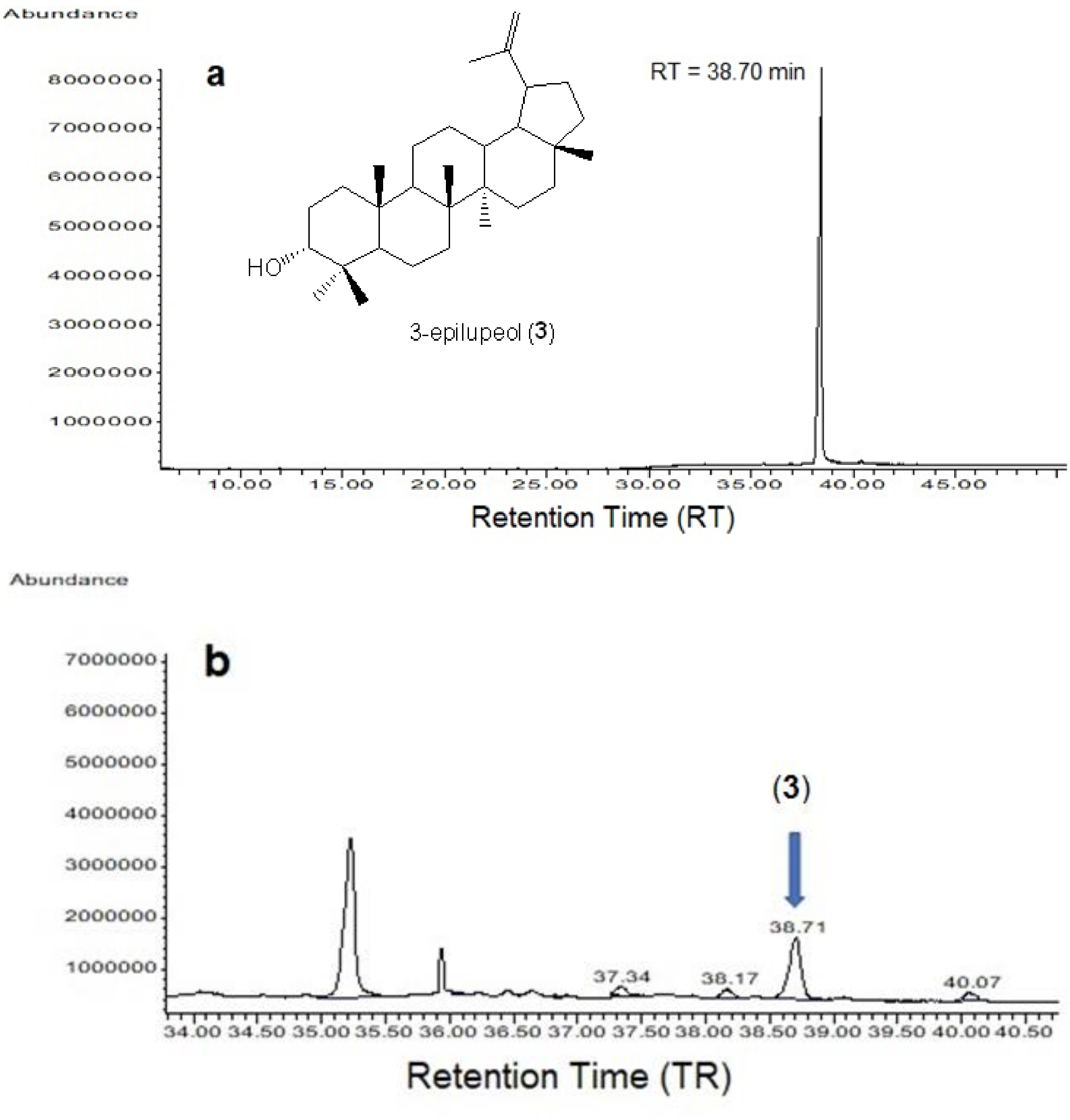

Figure S20. GC-MS chromatograms of standard compound and EtOAc extract. (**a**) 3-epilupeol (**3**) profile used as a standard; (**b**) EtOAc extract profile from *A. pichinchensis* cell suspension culture at 16 days of culture showing the peak of 3-epilupeol (**3**) compound.
